# Novel Non‐Cytotoxic Acylphosphinates and Acylphosphine Oxides Photoinitiators

**DOI:** 10.1002/anie.6797341

**Published:** 2026-05-25

**Authors:** Jiansong Yin, Yijun Zhang, Bernadette Graff, Céline Dietlin, Michael Schmitt, Fabrice Morlet‐Savary, Tatiana Petithory, Laurent Pieuchot, Jing Zhang, Xiaotong Peng, Yangyang Xu, Jean‐Michel Becht, Pu Xiao, Jacques Lalevée

**Affiliations:** ^1^ Université De Haute‐Alsace CNRS, IS2M UMR 7361 Mulhouse France; ^2^ Université De Strasbourg Strasbourg France; ^3^ Future Industries Institute University of South Australia Mawson Lakes Adelaide South Australia Australia; ^4^ State Key Laboratory of High Performance Ceramics Shanghai Institute of Ceramics Chinese Academy of Sciences Shanghai P. R. China; ^5^ College of Chemistry and Materials Science Anhui Normal University Wuhu P. R. China

**Keywords:** acylphosphine oxide and analogs, cytotoxicity, molecular design, photoinitiators, radical polymerization

## Abstract

This study focused on the design and synthesis of a series of novel acylphosphine oxide (APO) and acylphosphinate photoinitiators based either on diphenylphosphine oxide (DPO) or 9,10‐dihydro‐9‐oxa‐10‐phosphaphenanthrene 10‐oxide (DOPO), systematically investigating the structure–property relationships in terms of photochemical performance, polymerization efficiency and cytotoxicity. Polymerization experiments demonstrated that DPO‐based photoinitiators exhibit excellent photopolymerization efficiency, while the structurally optimized DOPO‐based Type I photoinitiators also exhibit good reactivity. Biocompatibility experiments indicate that acylphosphinates (e.g., the (6‐oxidodibenzo[c,e][1,2]oxaphosphinin‐6‐yl)(2,4,6‐trimethoxyphenyl)methanone (TMO‐DOPO) derivative) exhibit no significant toxicity toward C3H10 T1/2 mouse mesenchymal stem cells, significantly outperforming the traditional 2,4,6‐trimethylbenzoyldiphenylphosphine oxide (TPO) photoinitiator. These results highlight the DOPO role as a functional core in Type I initiation systems, providing an effective strategy for developing novel, efficient, and low‐toxicity photoinitiators.

## Graphical abstract

Novel acylphosphine oxide (APO) and acylphosphinate photoinitiators based on DPO and DOPO were designed to investigate structure–property relationships including photochemical performance, polymerization efficiency and cytotoxicity. While DPO derivatives showed excellent reactivity, DOPO‐based systems also displayed good efficiency and significantly lower toxicity than TPO, highlighting their potential as safer Type I photoinitiators.

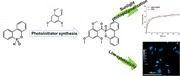

## 1 Introduction

Photopolymerization, as a green, efficient, and controllable polymerization technology [1], has garnered significant attention in industrial and research fields in recent years [2, 3]. The polymerization technique can be precisely controlled in both space and time through light exposure, with low energy consumption and no need for high temperatures [4, 5, 6, 7]. Currently, photopolymerization has been widely applied in fields such as ink coatings [8, 9], photoresists [10], 3D printing [11], biomaterials [12], dental materials [13], and optoelectronics [14], demonstrating significant advantages in enhancing production efficiency [15] and environmental sustainability [16].

Photoinitiators are the core components of photopolymerization processes [17, 18, 19], and their performance directly determines the polymerization conversion, curing depth, and the optical and mechanical properties of the final materials [20, 21]. Currently, widely used photoinitiating systems are based on monocomponent Type I photoinitiators that generate radicals directly through homolytic cleavage of a chemical bond upon light exposure [22]. Among these, phosphine oxide‐based photoinitiators have been extensively studied and widely applied in radical photopolymerization, as summarized in several comprehensive reviews on photoinitiator chemistry and mechanisms [23, 24, 25, 26]. In particular, acylphosphine oxides (APOs) have emerged as one of the most promising representatives of Type I photoinitiators due to their excellent absorption properties in the ultraviolet to near‐visible light spectrum, good photodecomposition ability, photobleaching behavior, and high initiation efficiency [27, 28]. For example, commercially available TPO and BPO (Phenylbis (2,4,6‐trimethylbenzoyl) phosphine‐oxide) are widely used in industrial light‐emitting diode (LED) curing systems. Upon light absorption, they undergo electronic transitions, followed by intersystem crossing (ISC) to the triplet state, where the C─P bond cleaves to generate two radicals—an acyl radical and a phosphorus‐centered radical—each capable of initiating polymerization.

Nevertheless, the widespread use of TPO and BPO is accompanied by strong concerning issues of cytotoxicity [28]. Existing research has shown that these photoinitiators and their photodegradation products may cause damage to cell membranes, induce oxidative stress, cause deoxyribonucleic acid (DNA) breaks, and even lead to cell apoptosis, posing potential hazards to biological systems [29, 30]. Therefore, the development of APO‐type photoinitiators that combine high photoinitiation efficiency with low cytotoxicity has become a core research direction but remains a huge challenge [31, 32]. Accordingly, rational molecular design must consider absorption wavelength, initiation efficiency, and thermal/light stability. In addition, ensuring end‐use safety is another important issue that future design efforts must prioritize [35, 36].

To this end, recent studies have attempted to optimize the structures of APO photoinitiators by varying either the acyl or the phosphinoyl group from a molecular structural perspective, thereby improving their photophysical properties and toxicity characteristics. For example, in our previous work, we introduced acyl groups bearing strongly electron‐donating aromatic substituents—namely, 2,4,6‐trimethoxybenzoyl (TMO) and 2,4‐dimethoxy‐6‐methylbenzoyl (DMO)—into APO photoinitiators [33], and this modification can effectively extend the absorption spectrum into the near‐visible light region, improve compatibility with common LED light sources, optimize photolysis behavior and radical generation pathways, and deliver robust photopolymerization efficiency.

In addition, 9,10‐dihydro‐9‐oxa‐10‐phosphaphenanthrene 10‐oxide (DOPO), a structurally unique phosphinate with an aromatic ring, exhibits excellent thermal stability [34, 37], biocompatibility, and dehydrogenation capability, making it a promising candidate for photopolymerization initiator design [28, 38]. In this respect, acylphosphinates have emerged as an interesting family of safer photoinitiators, particularly suitable for bio‐friendly light curing systems that are extremely sensitive to toxicity.

**FIGURE 1:**
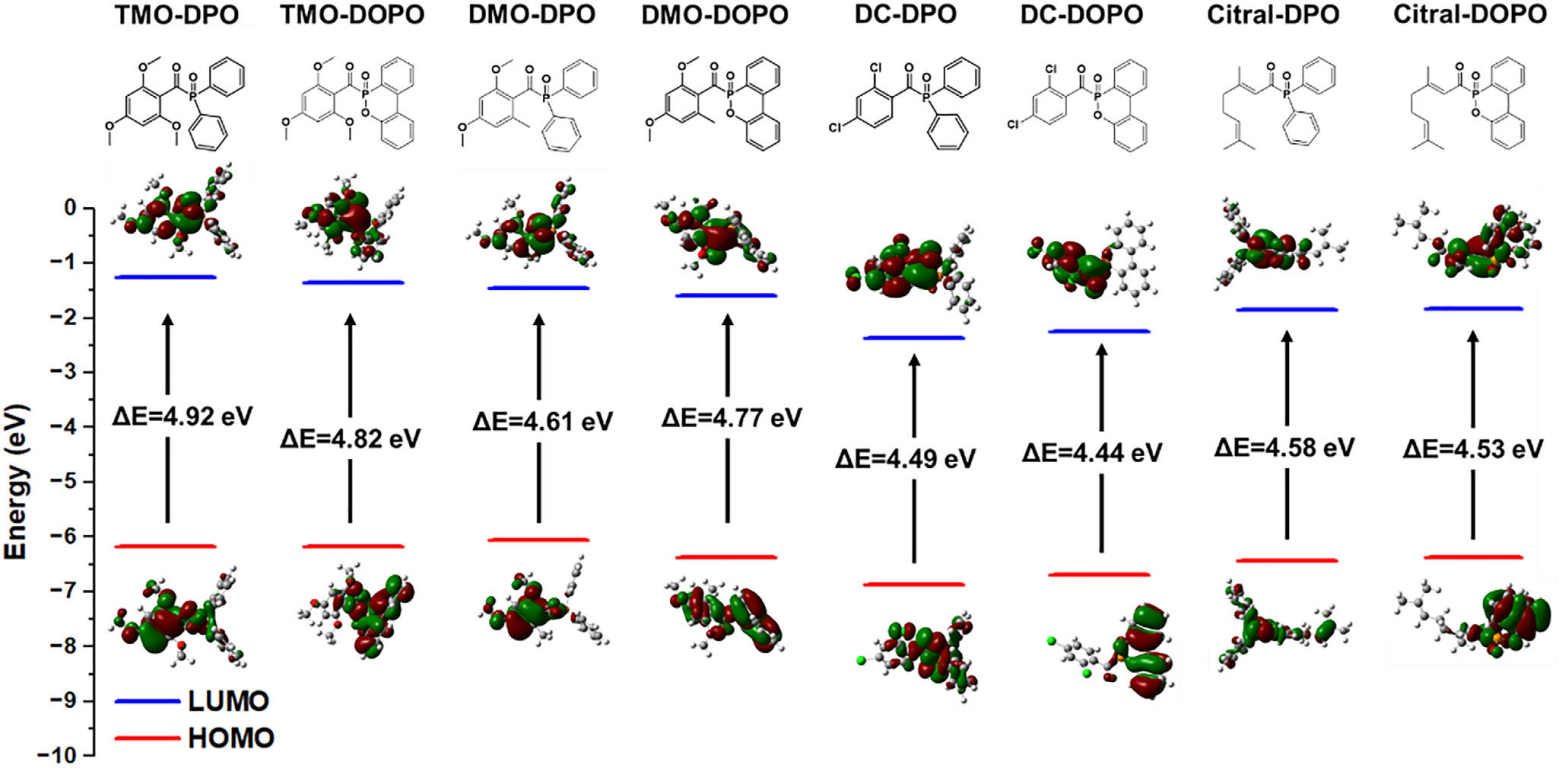
Molecular orbitals and energy level diagrams for the photoinitiators (calculation method: B3LYP/6‐31G(d) for geometry optimization and MPW1PW91/6‐31G(d) single point for orbital energies).

In this study, we explore both the effect of acyl and phosphine‐oxides moieties, that is, two previously reported photoinitiators [33] and six specifically newly designed photoinitiators were developed through DFT‐guided molecular design and subsequently synthesized, using either DPO or DOPO as the phosphine group and 2,4,6‐trimethoxybenzoyl, (2,4‐dimethoxy‐6‐methyl)benzoyl (DMO), or (2,4‐dichloro)benzoyl (DC) as the acyl groups. To further explore a biobased and sustainable design strategy, citral was also used as a natural, renewable starting material. As a proof‐of‐concept exploration, we further investigated the incorporation of a biosourced and safe fragment (citral) into the APO scaffold with the aim of reducing cytotoxicity. Although the resulting derivatives did not achieve performance comparable to the non‐biosourced analogues, this study provides valuable insights into the structure–property relationships governing this class of photoinitiators and highlights the challenges associated with balancing biosourced content with photoinitiating efficiency. Based on this, this study focuses on the synthesis of photoinitiators with tunable electronic properties, absorption characteristics, polymerization performance, and cytotoxicity (Figure 1). The results show that some of these compounds exhibit strong near‐UV absorption, favorable photochemical properties, and high initiation efficiency under 405 nm LED irradiation, while others can efficiently initiate polymerization even under sunlight irradiation, demonstrating significant potential for low‐energy‐consumption photopolymerization applications. Cytotoxicity assays based on live‐cell imaging revealed that a novel initiator (e.g., TMO‐DOPO) exerted weak effects on the proliferation and migration of mouse C3H10 T1/2 mesenchymal stem cells.

In summary, this study presents a series of photoinitiators that combine high performance with low toxicity through a molecular structure modulation strategy, providing both theoretical and experimental foundations for the development of the next‐generation high‐performance and environmentally friendly photoinitiators.

## 2 Results and Discussion

### 2.1 DFT Calculations

From the distribution of molecular frontier orbitals (Tables S1 and S2), the electron cloud of the highest occupied molecular orbital (HOMO) is mainly concentrated in conjugated regions such as aromatic rings and carbonyl groups, indicating that these orbitals are predominantly *π*‐type orbitals. Additionally, contributions from nonbonding orbitals (*n* orbitals) can also be observed in some structures, particularly in those containing P═O functional groups. For example, in DOPO‐based compounds (such as DC‐DOPO and citral‐DOPO), the HOMOs show partial electron density localized on the P═O moiety of the DOPO fragment, suggesting the participation of lone pair electrons derived from the P═O double bond in the DOPO fragment. The P═O group in DOPO may introduce an additional n‐orbital contribution, enhancing the possible *n*→π* component. The electron cloud distribution not only covers the aromatic ring region but also extends to the phosphorus‐oxygen functional group, indicating that it exhibits mixed orbital characteristics with partial *n*‐orbital contributions [28]. The involvement of this n orbital component may exert a certain influence on the transition type, leading to the occurrence of a partial *n*→π* transition [28].

The electron cloud distribution of the lowest unoccupied molecular orbital (LUMO) is more concentrated in the aromatic acyl group (such as 2,4,6‐trimethoxybenzoyl, 2,4‐dichlorobenzoyl) or phosphine oxide skeleton region, exhibiting distinct π* orbital characteristics [33]. These regions possess excellent conjugation properties, enabling them to efficiently accept electrons from the HOMO. In certain systems, such as DC‐DOPO, the electronic transitions exhibit distinct spatial separation: the HOMO is primarily localized on the DOPO moiety, while the LUMO is situated on the dichlorobenzoyl group, indicating a partial charge‐transfer transition.

In summary, the primary electronic transition is the *π*→π* transition, involving the transfer of electrons from *π* orbitals in electron‐rich regions to π* orbitals in the conjugated system, typically yielding strong absorption bands. However, for structures containing carbonyl or P═O groups, lone pair electrons introduce a certain *n*→π* character [28]. When the HOMO is partially localized on the P═O or C═O groups (e.g., DC‐DOPO, DMO‐DOPO), the contribution from *n*→π* transitions may become more significant.

The introduction of the DOPO moiety slightly influences the HOMO‐LUMO energy gap (Δ*E*); for example, DMO‐DPO exhibits Δ*E* = 4.61 eV, whereas DMO‐DOPO shows Δ*E* = 4.77 eV, indicating a slight increase with limited structural impact. The associated UV–vis spectra are given in Table S1. Finally, the efficient cleavage of the C(═O)─P covalent bond is crucial for achieving optimal performance as a Type I photoinitiator [28, 31, 33]. This requires favorable bond dissociation conditions under irradiation, governed by the balance between the bond dissociation energy (BDE) and the triplet excited‐state energy (*E*_T_) [33]. As calculated from the BDE and *E*_T_ values listed in Table S1, the bond dissociation enthalpies (Δ*H* = BDE—*E*_T_) are all close to or below zero, indicating that the cleavage reaction is energetically favorable from the triplet excited state [28].

### 2.2 Preparation and Stability Assessment

**SCHEME 1:**
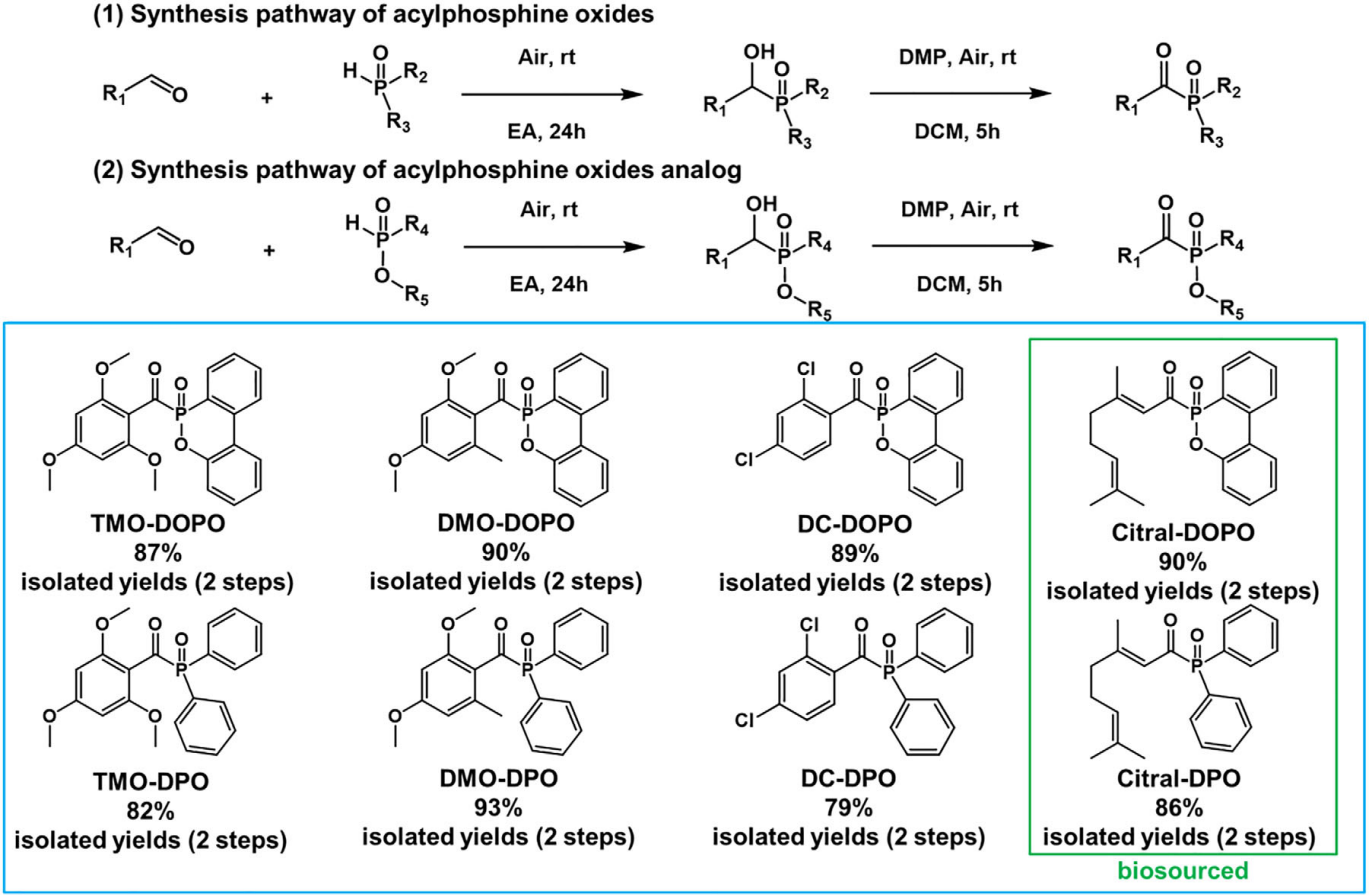
Synthetic route of DOPO and DPO derivatives.

The molecular design indicates that DOPO‐based photoinitiators hold significant potential, prompting their synthesis from commercially available aldehydes, including 2,4,6‐trimethoxybenzaldehyde, 2,4‐dimethoxy‐6‐methylbenzaldehyde, 2,4‐dichlorobenzaldehyde, and citral, and DOPO or DPO. The synthetic strategy involves the nucleophilic addition of DOPO or DPO to aldehydes, followed by oxidation to yield the corresponding APOs or acylphosphinates. This approach differs from the method reported in [36]. The resulting compounds (TMO‐DPO, DMO‐DPO, DC‐DPO, citral‐DPO, TMO‐DOPO, DMO‐DOPO, DC‐DOPO, and citral‐DOPO) are shown in Scheme 1 and Figures S1–S24. Among these, TMO‐DPO and DMO‐DPO serve as benchmarks, as previously reported [33]. Notably, using citral as a natural, renewable starting material, photoinitiators were successfully constructed in a simple two‐step procedure, highlighting a sustainable strategy for the development of bio‐based DOPO photoinitiators.

The stability of photoinitiators during storage has a significant impact on their potential practical applications. To assess this, stability tests were conducted on the synthesized photoinitiators. For this purpose, they were stored at room temperature in sealed, air‐filled containers, protected from light exposure. After 2 weeks, ^1^H‐NMR analysis was performed and compared with the ^1^H‐NMR spectra of the freshly synthesized APOs or analogs. It was observed that TMO‐DOPO and DMO‐DOPO were stable under these conditions, and no significant degradations were observed (see Figures S25–S27). This indicates that the storage conditions effectively maintained their molecular integrity during the testing period. However, DC‐DOPO, DC‐DPO, citral‐DOPO, and citral‐DPO showed noticeable changes after only 1 week under these conditions (see Figures S28–S31). Under the tested conditions, TMO‐DOPO, DMO‐DOPO, TMO‐DPO, and DMO‐DPO remained stable, whereas DC‐DOPO, DC‐DPO, citral‐DOPO, and citral‐DPO were unstable and were used in photopolymerizations directly after synthesis for further determination of their reactivity. Also, for these unstable compounds, DC‐DOPO, DC‐DPO, citral‐DOPO, and citral‐DPO, their structural integrity was verified by NMR prior to use.

### 2.3 Spectroscopic Characterization and Photochemical Reactivity of DOPO and DPO Derivatives

**FIGURE 2:**
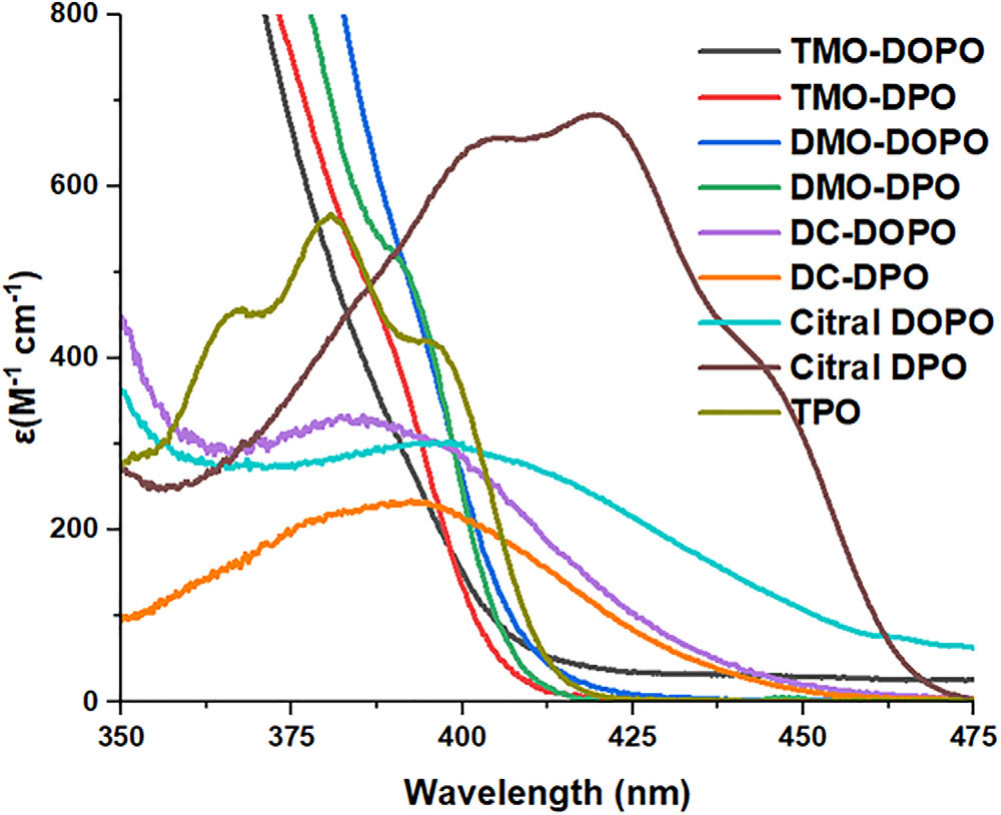
Molar extinction coefficient of photoinitiators in the range from 300 to 500 nm (in MeCN solvent).

The UV–vis absorption spectra of TPO and its derivatives were measured in acetonitrile (MeCN), and Figure 2 shows their absorption characteristics in the 300–500 nm wavelength range. Interestingly, the absorption bands of DC‐DOPO, DC‐DPO, citral‐DOPO, and citral‐DPO exhibit a red shift compared with that of TPO, particularly in Citral‐DPO, where the absorption maximum (*λ*_max_) shifts notably toward the longer wavelength region. Citral‐DPO/Citral‐DOPO introduces an *α*,*β*‐unsaturated aldehyde structure (citral group), which possesses a C═C─C═O conjugated system, enabling the formation of a large *π*‐delocalized network with the phosphine oxide moiety, slightly reducing the HOMO‐LUMO bandgap (Δ*E*) and causing the absorption wavelength to shift toward the visible light region. The conjugation effect stabilizes the excited state by extending the delocalized electron system, often causing a red shift in the *λ*_max_ [39].

By comparison, the absorption profiles of TMO‐DOPO, DMO‐DOPO, TMO‐DPO, and DMO‐DPO closely resemble those of TPO, showing no significant red shift. Their *λ*_max_ values are centered around approximately 385 nm, and at this wavelength, each compound exhibits a molar extinction coefficient similar to that of TPO. These findings suggest their strong potential as photoinitiators for near‐UV LED light sources. Additionally, the calculated absorption wavelengths (see Table S1) are consistent with the experimental spectral data (Figure 2), further validating the reliability of the quantum chemical calculations used to describe the excited‐state properties of APO‐type photoinitiators.

**FIGURE 3:**
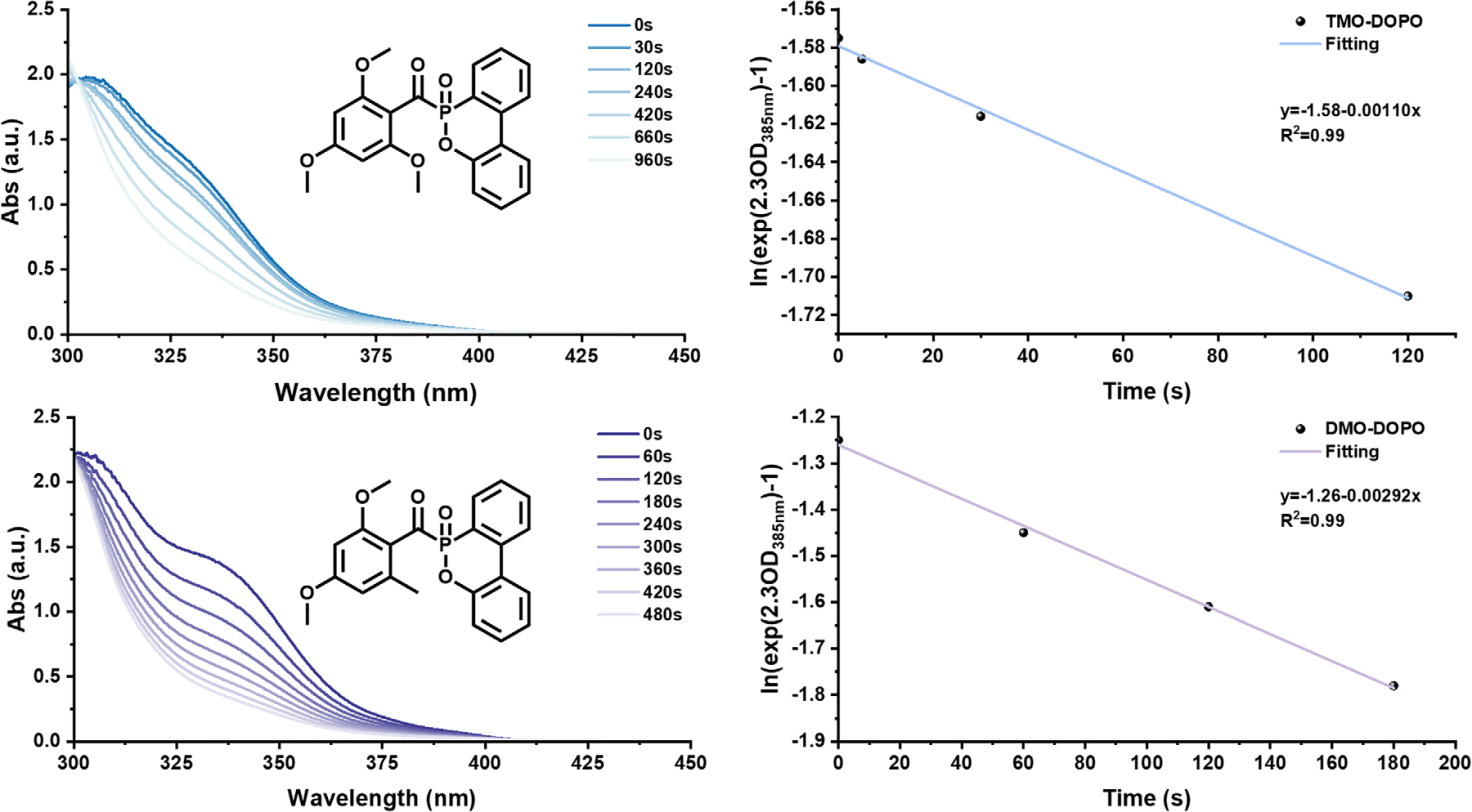
UV–vis absorption spectra recorded during steady‐state photolysis of the TMO‐DOPO and DMO‐DOPO under LED@385 nm irradiation.

Steady‐state photolysis studies were conducted in MeCN to investigate the photochemical behavior of the synthesized PIs and to gain a deeper understanding of their photolysis kinetic under LED illumination at 385 nm. Figures 3 and S32–S36 show that, within the 320–420 nm wavelength range, the UV–vis absorption intensity of the PI solutions gradually decreases with increasing irradiation time, indicating that the photoinitiators undergo continuous photolysis upon light exposure. Notably, no new absorption peaks were observed during the irradiation process, suggesting that the primary decomposition products of these photoinitiators do not exhibit significant light absorption in the 320–440 nm region. This result indicates that the radicals or other photoproducts generated by the cleavage of the P─C bond do not interfere with the original absorption characteristics of the photoinitiators in this wavelength range. To further quantify their photolysis behavior, the dissociation quantum yield (*Φ*) of each PI was estimated. This parameter reflects the efficiency of radical generation under irradiation at a specific wavelength and is an important indicator of photopolymerization efficiency.

To calculate the dissociation quantum yield, a linear fit of ln(exp(2.3 OD) – 1) versus time was performed [33]. The reference TPO is shown in Figure S32. All samples exhibited good first‐order photoreaction kinetics (Figures 3 and S33–36). Further calculations yielded dissociation quantum yields *Φ* of 0.05 for TMO‐DOPO and 0.08 for DMO‐DOPO. The *Φ* values for other photoinitiators are given in Table 1. Although the *Φ* values are relatively low (the value of *Φ*_TPO_ was 0.56 [40]), the UV–vis absorption and promising theoretical calculation results prompted us to conduct further characterizations. Indeed, the cleavage quantum yield alone does not dictate practical photoinitiating performance. The reactivity of the generated radicals is also a key point, as well as their potential side reactions. While DC‐DPO exhibited a much higher *Φ* of 0.85. In contrast, DC‐DOPO, citral‐DPO, and citral‐DOPO showed relatively low *Φ* values of 0.01, 0.07, and 0.04, respectively. The cleavage quantum yields of TMO‐DPO and DMO‐DPO have been previously reported as 0.54 and 0.57, respectively [33]. In our experiments, the calculated values were 0.54 and 0.55, showing good agreement with the literature.

**TABLE 1:** Photolysis quantum yield of photoinitiators (in solvent MeCN).

	TMO‐DOPO	DMO‐DOPO	DC‐DPO	DC‐DOPO	Citral‐DPO	Citral‐DOPO
*Φ*	0.05	0.08	0.85	0.01	0.07	0.04

### 2.4 Radical Polymerization Experiments of TMPTA

**FIGURE 4:**
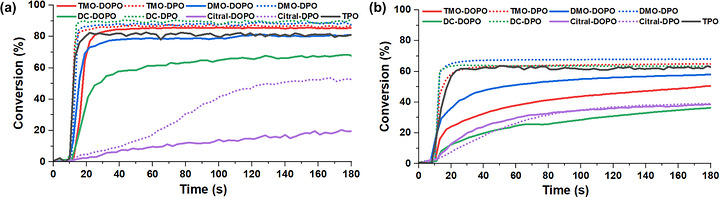
Photopolymerization kinetics of TMPTA with photoinitiators (PIs: 2.80×10^−5^mol·g^−1^) under 405 nm LED irradiation: (a) in a thick sample (∼2 mm) and (b) in a thin film (∼25 µm). Irradiation starts at *t* = 10 s.

The photopolymerization efficiency of TMPTA was evaluated in both thick samples (∼2 mm) and thin films (∼25 µm) to assess the impact of molecular structure on curing performance. In the present study, the photochemical quantum yields were determined using a 385 nm LED, which is commonly employed in photoinitiator investigations and allows direct comparison with literature data. It should be noted that the photochemical reactivity of photoinitiators may depend on the irradiation wavelength. In several photopolymerization systems, a red‐shift of photochemical reactivity relative to the absorption spectrum has been reported, meaning that efficient photoinitiation can occur even at wavelengths where the absorption coefficient is relatively low. [28, 29, 30, 31, 32, 33, 34, 35, 36, 37, 38, 39, 40, 41, 42] In this context, the photoinitiation ability of the present systems was further evaluated through photopolymerization experiments under 405 nm LED irradiation but also under sunlight (see below). For thick samples, DC‐DPO exhibited the highest final conversion (≈90%) and an extremely short induction period, initiating polymerization rapidly upon light exposure and quickly reaching the maximum conversion, demonstrating its unique initiation capability. The purity and integrity of DC‐DPO were verified prior to use to ensure that the observed performance reflects its inherent photoinduction efficiency. The polymerization conversions of the photoinitiators are ranked as follows: DC‐DPO > DMO‐DPO ≈ TMO‐DPO ≈ TMO‐DOPO > TPO ≈ DMO‐DOPO > DC‐DOPO > citral‐DPO > citral‐DOPO (see Figure 4a). Among these, the previously reported DMO‐DPO and TMO‐DPO exhibit excellent performance, achieving polymerization conversions of approximately 80%, confirming their strong initiating ability under high optical path length conditions. The newly synthesized DC‐DPO outperforms these known initiators. TMO‐DOPO and TMO‐DPO exhibit similar kinetic curves and induction periods, indicating that the introduction of DOPO does not significantly impair their initiating ability in thick samples. Under these conditions, the polymerization efficiency of citral‐based initiators is relatively low, possibly due to their lower radical yields or less reactive acyl radicals [45].

In thin samples (Figure 4b), the ranking of initial efficiency slightly changes: DMO‐DPO > TMO‐DPO ≈ DC‐DPO ≈ TPO > DMO‐DOPO > TMO‐DOPO > citral‐DPO ≈ citral‐DOPO > DC‐DOPO. Under these shorter optical path conditions, DMO‐DPO and TMO‐DPO maintain strong initiation efficiencies, with DMO‐DPO ranking first and achieving a polymerization conversion of approximately 65%, highlighting its multifunctional structural advantages across different curing environments. DC‐DPO also exhibits excellent efficiency in thin films, with an extremely short induction period and rapid polymerization onset, quickly reaching the maximal conversion of approximately 60%. Additionally, the polymerization efficiencies of citral‐DPO and citral‐DOPO are comparable, both achieving conversions of approximately 40%, suggesting that for non‐aromatic structures, initiation limitations may primarily stem from their inherent photoreactive properties rather than differences in phosphine oxide molecules.

A comprehensive analysis of the polymerization behavior of thick and thin film samples reveals that the molecular structure of the initiator, particularly the type and position of substituents, significantly influences its performance at different curing depths. Aromatic substituents enhance light absorption and radical efficiency, demonstrating stable advantages in both thick and thin films. Meanwhile, the incorporation of the DOPO group maintains a certain level of initiation performance while potentially imparting additional functionalities, such as thermal stability and flame retardancy, to the system [34]. Additionally, recent studies have indicated that introducing DOPO groups into the structure of photoinitiators can reduce their cytotoxicity [28]. Through rational structural optimization tailored to specific application requirements, the practical value of these photoinitiators can be further enhanced.

Although DC‐DPO exhibits exceptionally high initiation efficiency in photopolymerization experiments, its stability in the neat form is limited, with noticeable degradation observed after 2 weeks of storage at room temperature. Given its outstanding polymerization conversion rate, we further evaluated its practical stability in application‐relevant environments, specifically its storage behavior within monomer formulation systems. Specifically, a TMPTA monomer system containing DC‐DPO was sealed, protected from light, and stored at room temperature in air for 2 months before undergoing photopolymerization testing. Results showed that the polymerization efficiency of the 2‐month‐stored sample under LED@405 nm conditions was nearly identical to that of a freshly prepared sample, with conversion remaining essentially consistent at approximately 90% (see Figure S37). This finding indicates that despite the high reactivity of DC‐DPO, the monomer system effectively suppresses its spontaneous degradation, providing a storage and handling window for premixed formulations. This further highlights the application potential of DC‐DPO as a highly efficient photoinitiator, demonstrating that the instability of its free molecules can be overcome through appropriate storage and formulation strategies, enabling stable and efficient initiation in photopolymerization systems.

### 2.5 Sunlight Photopolymerization

Given that the synthesized photoinitiators exhibit excellent photoresponsiveness under LED excitation at 405 nm, further exploration of their application potential under visible light sources, particularly natural light (such as sunlight), is of particular importance. Sunlight offers unique advantages as a low‐cost and renewable light source [46, 47, 48]. Therefore, based on their radical generation capabilities, the photopolymerization behavior of representative photoinitiators under sunlight irradiation was further investigated to assess their curing efficiency and practical feasibility in the absence of artificial light sources.

In sunlight‐induced polymerization experiments, the performance of different initiators generally followed the relative trends observed under artificial light sources (LED@405 nm) but also exhibited certain differences in light source adaptability. The polymerization efficiency, from highest to lowest, was DC‐DPO > DMO‐DPO ≈ TMO‐DPO ≈ TMO‐DOPO ≈ TPO ≈ DMO‐DOPO > DC‐DOPO > citral‐DPO > citral‐DOPO. Once again, DC‐DPO exhibited the highest polymerization efficiency, achieving a final conversion of approximately 80%. DMO‐DPO and TMO‐DPO, as previously reported high‐performance initiators, also maintain stable initiation activity under sunlight, achieving polymerization conversions of approximately 60%. Their structurally similar counterparts, TMO‐DOPO and DMO‐DOPO, exhibiting similar performances.

**FIGURE 5:**
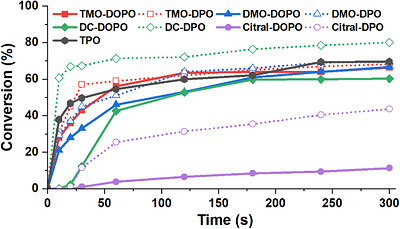
Photopolymerization kinetics of TMPTA with photoinitiators (PIs: 2.80×10^−5^mol·g^−1^) under 405 nm LED irradiation in a thick sample.

It is worth noting that the efficiency of the traditional commercial initiator TPO under sunlight is comparable to that of certain DOPO derivatives (such as TMO‐DOPO and DMO‐DOPO), both achieving polymerization conversions of approximately 60%. This indicates that these novel molecules exhibit good performance compared with TPO. The polymerization profiles of DC‐DOPO and citral‐based initiators are relatively lower. Overall, most DPO and DOPO‐derived photoinitiators can achieve effective polymerization under sunlight conditions, with DC‐DPO reaching approximately 80% conversion and both DMO‐DPO and TMO‐DPO around 60%, demonstrating their good performance (details in Figure 5). These results reveal a robust photochemical reactivity that transcends the absorption maximum, facilitating the use of various light sources [41]. Such versatility paves the way for their implementation in green energy, outdoor coatings, and environmentally friendly photopolymerization. In the present study, although photolysis quantum yield measurements were only performed at 385 nm, photopolymerization under 405 nm LED irradiation and natural sunlight demonstrated that the developed photoinitiators remain active under longer and broad‐spectrum wavelengths. These observations suggest that the systems may retain high efficiency on the red side of the absorption spectrum, allowing polymerization under conditions of low absorption, deep penetration, and minimal photodamage, which is particularly attractive for biological and environmentally friendly applications.

The observed photopolymerization efficiency of the developed photoinitiators is closely linked to the interplay between molecular structure, electronic properties, and radical generation capability. DFT calculations indicate that variations in the acyl and phosphine oxide moieties modulate the HOMO–LUMO gap and the distribution of frontier orbitals, which in turn affect light absorption and the likelihood of C(═O)─P bond cleavage. For example, DOPO‐modified photoinitiators exhibit partial *n*→π* character due to the P═O lone pair electrons, which can influence the efficiency of radical formation under near‐UV and visible light. Real‐time photolysis experiments and EPR spin‐trapping confirm that these structural modifications lead to differences in radical generation rates and radical types. Consequently, the efficiency of TMPTA polymerization under both 405 nm LED and natural sunlight irradiation reflects the combined effects of absorption properties, photolysis behavior, and radical reactivity, providing a coherent structure–reactivity–performance relationship.

### 2.6 Electron Paramagnetic Resonance (EPR) Experiment

**FIGURE 6:**
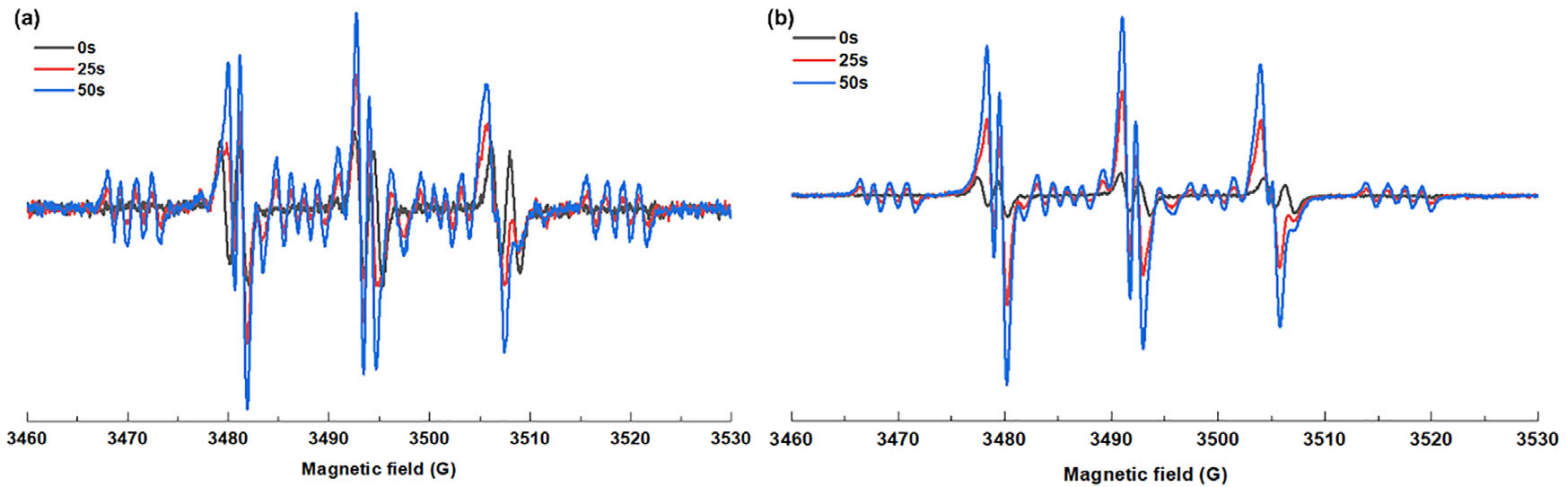
The PBN radical adducts (EPR spectra using *tert*‐butylbenzene as a solvent). The spectra detected at 0, 25, and 50s upon irradiation with LED@405 nm (a) TMO‐DOPO and (b) DMO‐DOPO.

**SCHEME 2:**
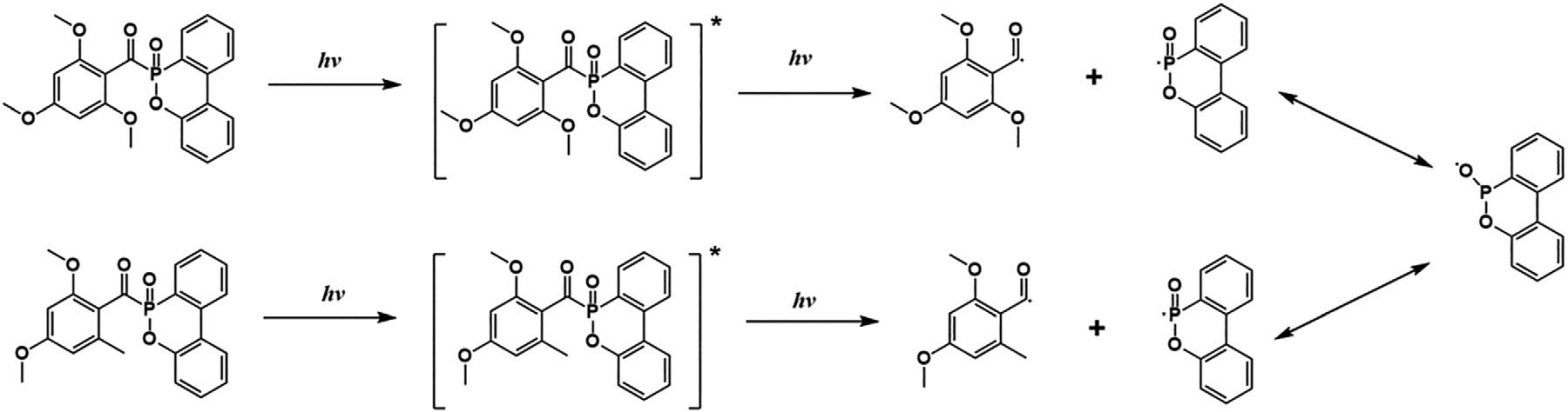
Proposed photofragmentation mechanism of TMO‐DOPO and DMO‐DOPO.

As DOPO derivatives being much less studied than DPO derivatives, the potentially reactive radicals generated through C─P bond cleavage of DOPO derivatives under 385 nm LED irradiation were then investigated by EPR experiments (Figure 6) [39, 49]. Both TMO‐DOPO and DMO‐DOPO exhibited distinct radical signals under dark conditions (see Figure 6). However, the experimental results indicate that they cannot effectively initiate monomer polymerization in the dark, with polymerization conversion remaining close to zero. After illumination, TMO‐DOPO and DMO‐DOPO generate three types of radicals: a carbon‐centered radical, a phosphorus‐centered radical, and an oxygen‐centered radical [28]. The possible radical generation mechanism is illustrated in Scheme 2 [28].

In addition, as detailed in the Supporting Information (DOPO as a hydrogen donor), the role of the P─H bond in DOPO as a potential Type II hydrogen donor was investigated, providing complementary insights into its photochemical reactivity (Figures S38–S41). EPR analyses confirmed that DOPO can spontaneously generate oxygen‐centered radicals and participate in hydrogen‐transfer processes with a typical Type II photoinitiator, such as isopropyl‐thioxanthone (ITX). Photophysical and photopolymerization experiments further demonstrated that DOPO enhances the photolysis of ITX and effectively promotes radical formation, leading to improved polymerization efficiency—particularly under thick‐film conditions—compared with the traditional ITX/ethyldimethylaminobenzoate (EDB) system. Moreover, when paired with Couma 3 (a compound previously reported by our group [50]), DOPO significantly shortens the induction period and yields faster polymerization than the conventional EDB‐based system, highlighting its superior performance as a hydrogen donor. Collectively, these results confirm that DOPO can function as an efficient hydrogen‐donor component in Type II photoinitiation systems.

### 2.7 Evaluation of Photoinitiator Cytotoxicity

TPO has been widely reported to exhibit cytotoxic behavior [28, 29, 30], which has, to some extent, limited its further application in bio‐related fields. DC‐DPO is highly efficient for photopolymerization, but it is not highly stable in its neat form. Therefore, to explore the potential for improving cellular compatibility through molecular structure optimization, this study evaluated the cytotoxicity profile of the novel DOPO derivative TMO‐DOPO. TMO‐DOPO was chosen for comparison with TPO because its structurally optimized design provides improved storage stability while maintaining high photopolymerization efficiency comparable to that of TPO. This comparative analysis enables the evaluation of potential improvements in cellular compatibility resulting from structural optimization while preserving photoinitiation efficiency, providing a reference for its potential application in bio‐related fields. The HoloMonitor M4 label‐free live cell imaging system was used to dynamically track the migration and proliferation behavior of mouse C3H10 T1/2 mesenchymal stem cells treated with TMO‐DOPO and TPO. The monitoring was conducted over an 18‐h period, which effectively captures the critical window for acute cellular toxicity immediately following exposure—a standard timeframe for assessing the short‐term safety of biomedical curing systems. All experiments were conducted at a uniform concentration of 50 µM, as it is well‐documented in the literature that TPO exhibits significant cytotoxicity at this specific concentration [28].

**FIGURE 7:**
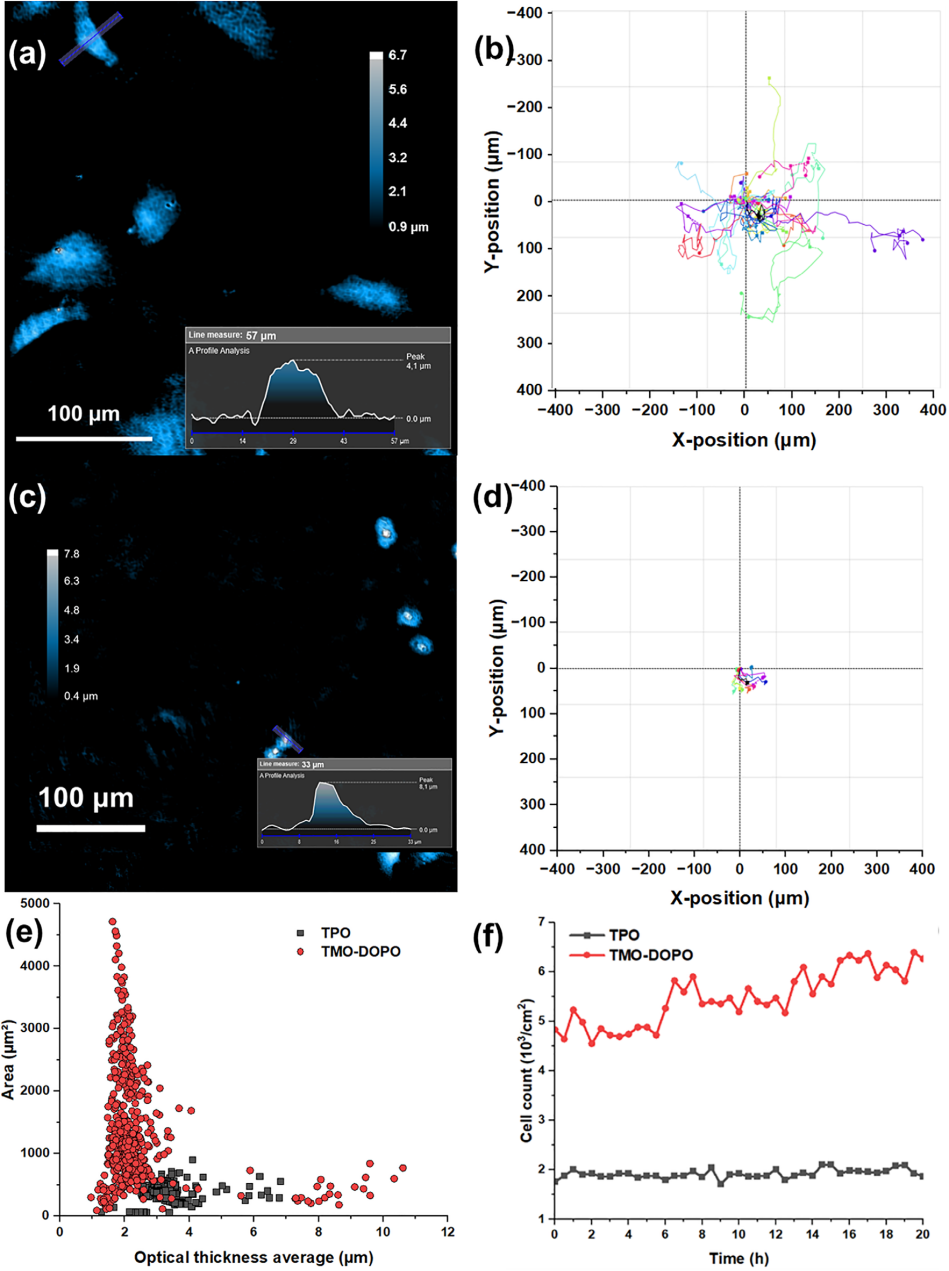
Holographic phase images of C3H10 T1/2 cells treated with (a) TMO‐DOPO and (c) TPO at random locations (bottom‐right insets show profile analysis, (c) reproduced from [28]). Single‐cell tracking images of the experimental groups treated with (b) TMO‐DOPO and (d) TPO, (d) reproduced from [28]. (e) Analysis of cell morphology. (f) Comparison of cell counts between TPO and TMO‐DOPO treatment groups.

The results showed that cells in the TPO‐treated group rapidly lost their migration and proliferation capabilities within a short time (Figures 7c,d,f and S42; Videos S1 and S2), with most cells adopting a rounded morphology and some cells detaching from the substrate, indicating cell death. In contrast, the TMO‐DOPO‐treated group exhibited markedly different behavior: cell numbers increased steadily over time, and migration pathways remained active (Figures 7a,b,f and S42; Videos S3 and S4), indicating that cells maintained normal proliferative activity. Further morphological analysis revealed that the cells were in good condition, exhibiting a well‐spread morphology without rounding or contraction (Figure 7e), further supporting their excellent biocompatibility.

Regarding the potential impact of photolysis fragments in real‐life applications, it is crucial to consider the specific photochemical behavior of APO‐based systems. During the photolysis and photocleavage reactions, initiators like TPO and our DOPO derivatives undergo *α*‐cleavage to generate highly reactive phosphinoyl and acyl radicals. Rather than accumulating as free fragments via hydrogen abstraction or recombination, these primary radicals predominantly react with monomers and become covalently incorporated into the polymer network, forming unextractable structures [51]. Consequently, the primary biological risk in practical applications is associated with unreacted photoinitiator that remains extractable from the cured matrix. Therefore, the toxicity of the neat, unreacted molecule is of paramount concern. By demonstrating that the structurally optimized TMO‐DOPO exhibits significantly lower cytotoxicity than the neat TPO, this study directly addresses the most critical factor governing the biosafety of these photoinitiating systems.

In summary, the results indicate that introducing DOPO groups to modify the TPO backbone significantly reduces its cytotoxicity and improves its biocompatibility under physiological conditions. This molecular modification strategy provides an effective approach for developing photoinitiators that combine photoinitiation performance with biosafety, offering potential applications in biomaterials, where stringent toxicity thresholds must be met, as well as in environmentally friendly green curing systems. These insights highlight the potential of rational molecular design in tuning photoinitiator reactivity while maintaining biocompatibility, offering guidance for future development of high‐performance and low‐toxicity photoinitiating systems.

## 3 Conclusion

In this study, a series of novel APO and acylphosphinate photoinitiators were designed and synthesized, including several derivatives obtained from DPO and DOPO, with the introduction of acyl substituents such as TMO, DMO, DC, and a citral‐derived group. The photochemical properties of these initiators were systematically evaluated by means of theoretical calculations, UV–vis absorption spectroscopy, steady‐state photolysis, and polymerization experiments. It was found that the introduction of the DOPO structure could effectively modulate the molecular orbital energy level, reduce Δ*E*, and moderately red‐shift the absorption spectrum while maintaining good initiation activity. In addition, the results of biocompatibility experiments showed that the representative initiator, TMO‐DOPO, was not significantly toxic to mouse mesenchymal stem cells and maintained normal cell migration and proliferation, which was significantly better than that observed for the conventional TPO photoinitiator. These findings highlight the pivotal role of the DOPO as the functional core in the molecular design of safe Type I photoinitiators, providing novel design strategies and a theoretical foundation for developing highly efficient and non‐toxic next generation photoinitiators. Overall, this research demonstrates significant scientific value and promising application prospects.

## Author Contributions

**Jiansong Yin**: data curation, writing – original draft. **Yijun Zhang**: writing – review and editing, validation. **Bernadette Graff**: data curation, writing – review and editing. **Céline Dietlin**: data curation, writing – review and editing. **Michael Schmitt**: data curation, writing – review and editing. **Fabrice Morlet‐Savary**: data curation, writing – review and editing. **Tatiana Petithory**: data curation, writing – review and editing. **Laurent Pieuchot**: data curation, writing – review and editing. **Jing Zhang**: conceptualization, methodology, validation, writing – review and editing, investigation. **Xiaotong Peng**: methodology, writing – review and editing. **Yangyang Xu**: methodology, validation, writing – review and editing. **Jean‐Michel Becht**: methodology, validation, writing – review and editing. **Pu Xiao**: conceptualization, investigation, methodology, validation, writing – review and editing. **Jacques Lalevée**: conceptualization, investigation, methodology, validation, writing – review and editing.

## Conflicts of Interest

The authors declare no conflicts of interest.

## Supporting information




**Supporting File 1**: anie72889‐sup‐0001‐SuppMat.docx.

**Supporting File 2**: anie72889‐sup‐0002‐videoS1.mp4.

**Supporting File 3**: anie72889‐sup‐0003‐videoS2.mp4.

**Supporting File 4**: anie72889‐sup‐0004‐videoS3.mp4.

**Supporting File 5**: anie72889‐sup‐0005‐videoS4.mp4.

## Data Availability

The data that support the findings of this study are available from the corresponding author upon reasonable request.
