# Supplementary material for: Novel Non‐Cytotoxic Acylphosphinates and Acylphosphine Oxides Photoinitiators
Source: Angew Chem Int Ed Engl. 2026 May 25;65(30):e6797341. doi: 10.1002/anie.6797341 (PMC13383056; doi:10.1002/anie.6797341)
Supplement: Supplementary file 1 — Supporting File 1: anie72889‐sup‐0001‐SuppMat.docx. [file ANIE-65-e6797341-s003.docx]

**Supporting Information**

**Novel Non-Cytotoxic Acylphosphinates and Acylphosphine Oxides Photoinitiators**

Jiansong Yin^a,b^, Yijun Zhang^a,b^, Bernadette Graff^a,b^, Céline Dietlin^a,b^, Michael Schmitt^a,b^, Fabrice Morlet-Savary^a,b^, Tatiana Petithory^a,b^, Laurent Pieuchot^a,b^, Jing Zhang^c^, Xiaotong Peng^e^, Yangyang Xu^d^*, Jean-Michel Becht^a,b*^, Pu Xiao^e^* and Jacques Lalevée^a,b*^

^a^Université de Haute-Alsace, CNRS, IS2M UMR 7361, F-68100 Mulhouse, France.

^b^Université de Strasbourg, France.

^c^Future Industries Institute, University of South Australia Mawson Lakes, SA 5095, Australia

^d^College of Chemistry and Materials Science, Anhui Normal University, South Jiuhua Road 189, Wuhu 241002, P. R. China.

^e^State Key Laboratory of High Performance Ceramics, Shanghai Institute of Ceramics, Chinese Academy of Sciences, Shanghai 200050, P. R. China

**Theoretical computations and molecular modeling**

Calculations rely on Density Functional Theory (DFT) using the Gaussian software. Ground state and triplet state geometries have been optimized for the APOs studied in this paper and for TPO used as a comparison at the UB3LYP/6-31G(d) level of theory. The same is true for the radicals used for the bond dissociation energy (BDE) calculations and for the spin density computation. The lowest unoccupied molecular orbital (LUMO) and the highest occupied molecular orbital (HOMO) listed in Figure 1 (The enlarged version is provided in Table S2.) and Table S1 have been computed in a single point at a RTD-mPW1PW91-FC/6-31G* level of theory on the previously optimized geometries.

Table S1. Theoretical calculations method used (B3LYP/6-31G*)

| Optimized  structures | BDE  (kcal mol^-1^) | E_T_  (kcal mol^-1^) | Spectra UV-vis |
| --- | --- | --- | --- |
| 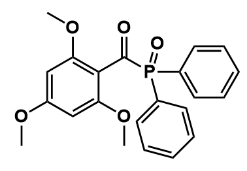  TMO-DPO | 57.5 | 59.7 | 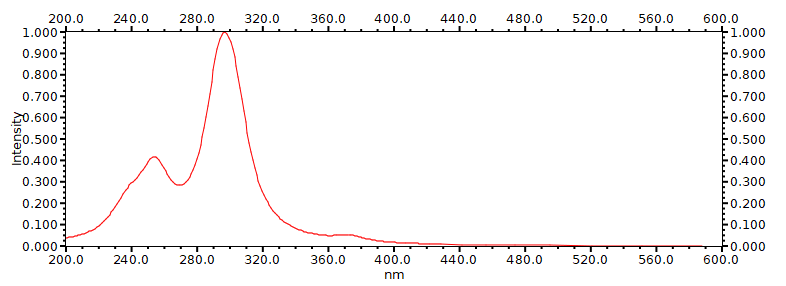 λ_max_ = 373.21 nm F = 0.0075  λ_max_ = 303.09 nm F = 0.1340  λ_max_ = 294.01 nm F = 0.1886  λ_max_ = 254.92 nm F = 0.0112 |
| 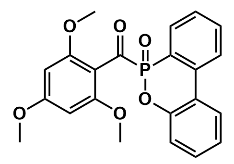  TMO-DOPO  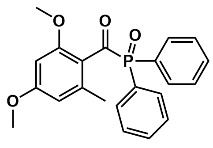  DMO-DPO  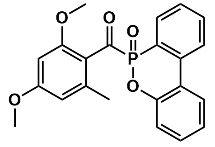  DMO-DOPO  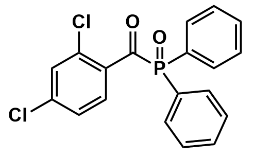  DC-DPO  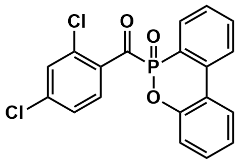  DC-DOPO  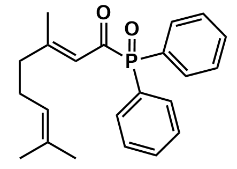  Citral-DPO  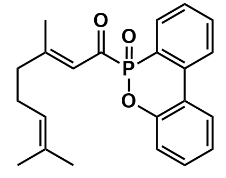  Citral-DOPO | 55.8  54.0  53.5  59.8  60.4  64,4  58,4 | 54.4  56.5  60.3  52.2  52.7  67.8  53.1 | 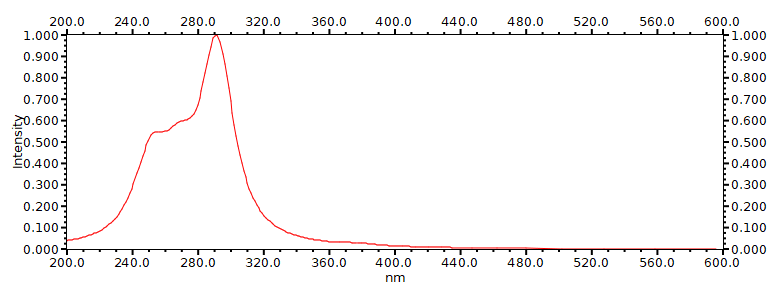  λ_max_ = 378.20 nm F = 0.0030  λ_max_ = 295.48 nm F = 0.0386  λ_max_ = 293.82 nm F = 0.1093  λ_max_ = 289.60 nm F = 0.1675  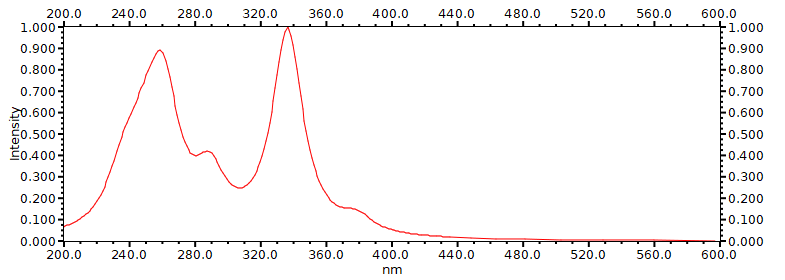  λ_max_ = 379.05 nm F = 0.0106  λ_max_ = 336.62 nm F = 0.1434  λ_max_ = 289.32 nm F = 0.0365  λ_max_ = 262.60 nm F = 0.0216  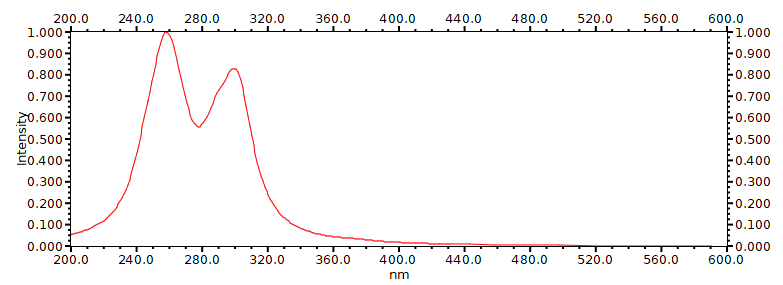  λ_max_ = 374.93 nm F = 0.0016  λ_max_ = 303.77 nm F = 0.0810  λ_max_ = 299.74 nm F = 0.0803  λ_max_ = 289.57 nm F = 0.0151  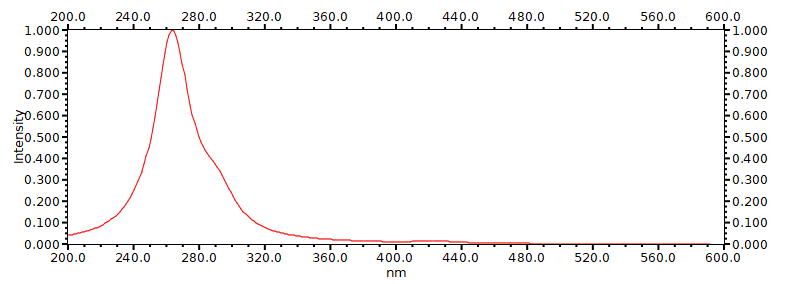  λ_max_ = 424.22 nm F = 0.0050  λ_max_ = 292.87 nm F = 0.0312  λ_max_ = 288.84 nm F = 0.0542  λ_max_ = 274.92 nm F = 0.0130  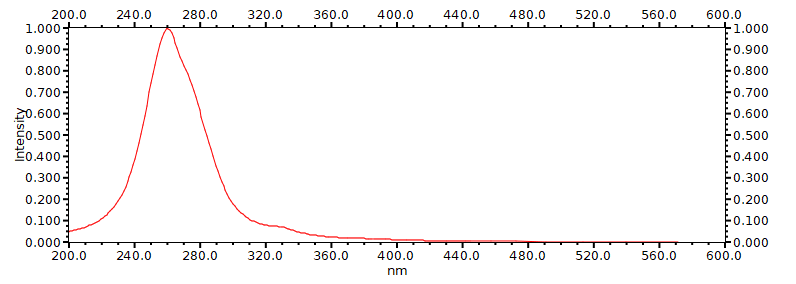  λ_max_ = 386.93 nm F = 0.0020  λ_max_ = 330.09 nm F = 0.0133  λ_max_ = 289.91 nm F = 0.0151  λ_max_ = 283.31 nm F = 0.0708  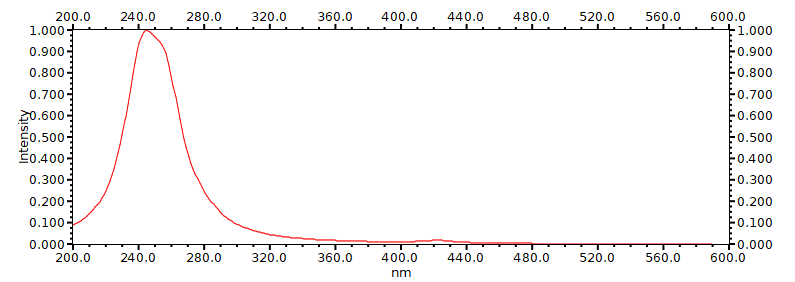  λ_max_ = 422.69 nm F = 0.0061  λ_max_ = 280.95 nm F = 0.0177  λ_max_ = 259.00 nm F = 0.0154  λ_max_ = 256.78 nm F = 0.2710  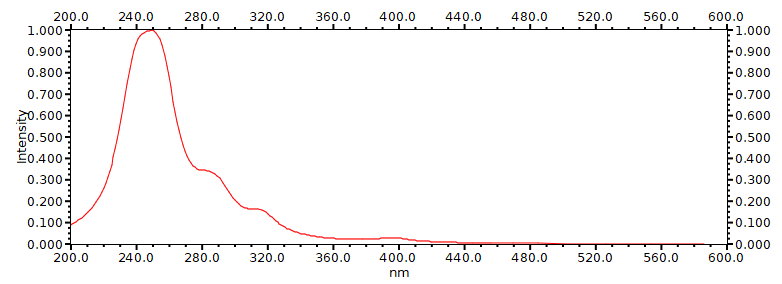  λ_max_ = 396.89 nm F = 0.0083  λ_max_ = 316.72 nm F = 0.0374  λ_max_ = 290.86 nm F = 0.0492  λ_max_ = 283.43 nm F = 0.0432 |

Table S2. Optimized structures, HOMO and LUMO of PIs and TPO. Theoretical calculations method used (B3LYP/6-31G*)

| Optimized  structures | HOMO | LUMO |
| --- | --- | --- |
| 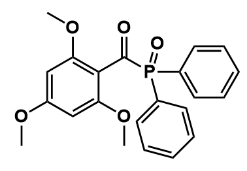  TMO-DPO | 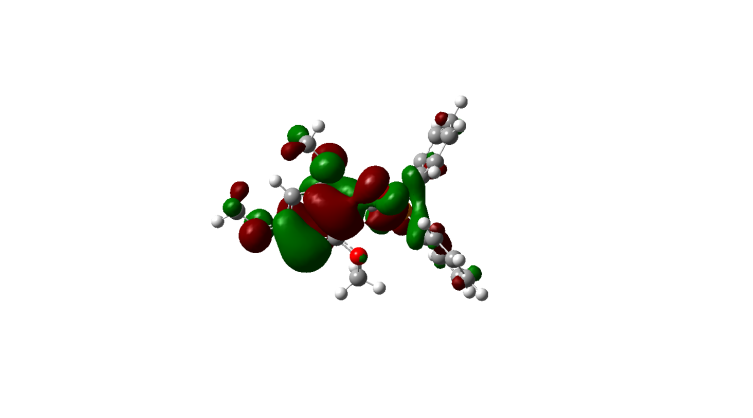 | 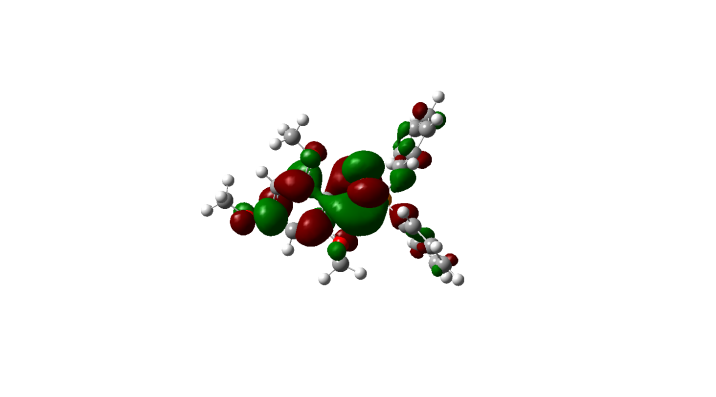 |
| 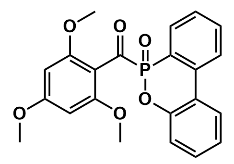  TMO-DOPO  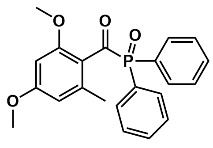  DMO-DPO  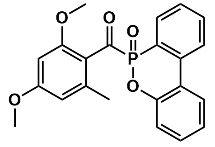  DMO-DOPO  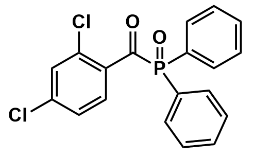  DC-DPO  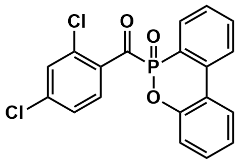  DC-DOPO  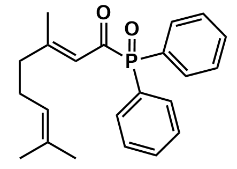  Citral-DPO  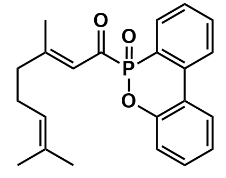  Citral-DOPO | 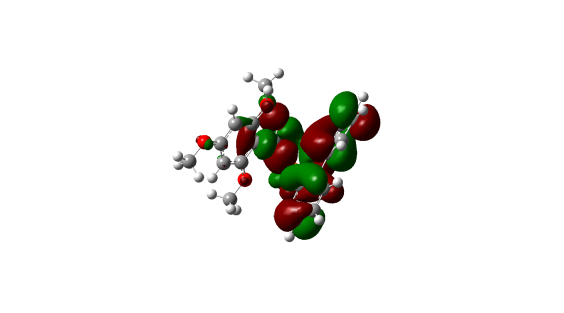  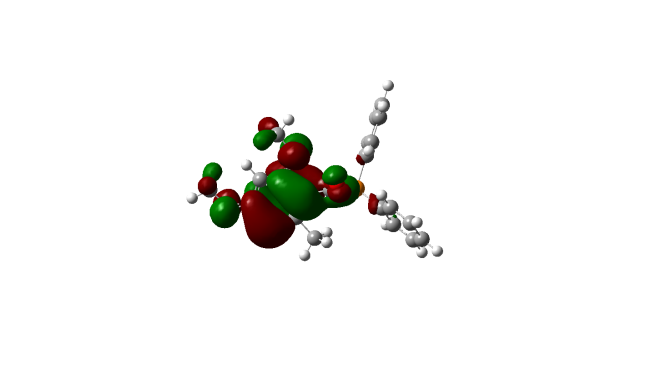  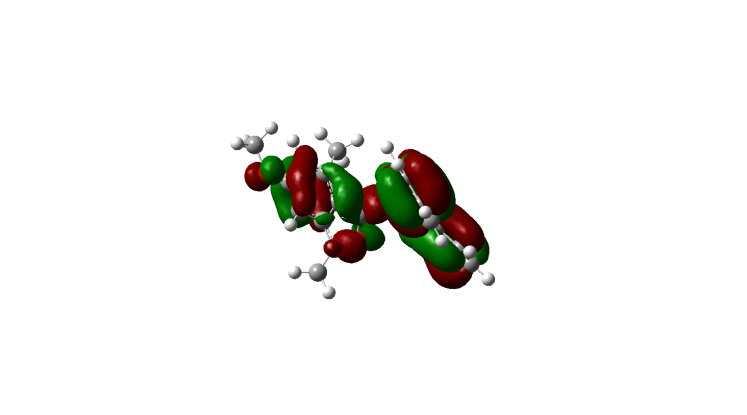  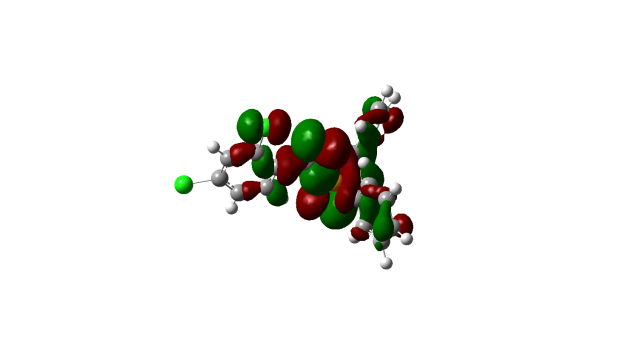  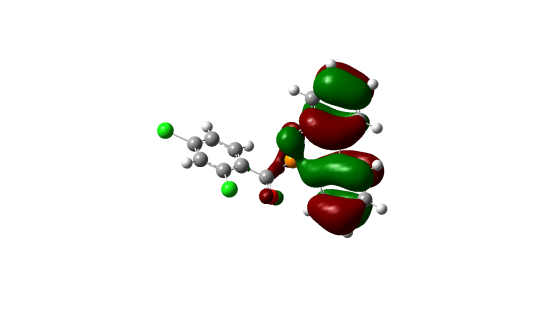  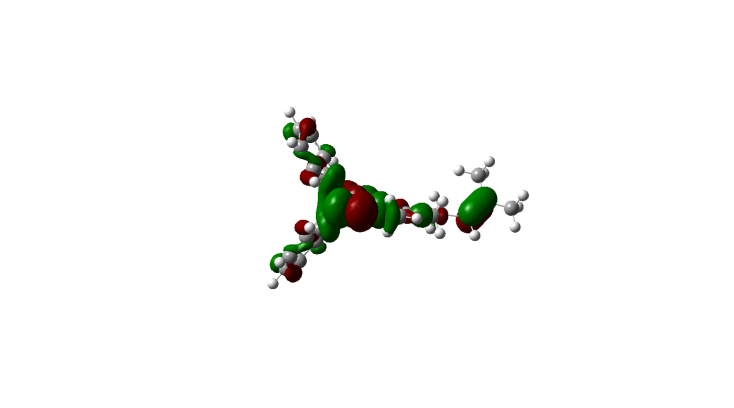  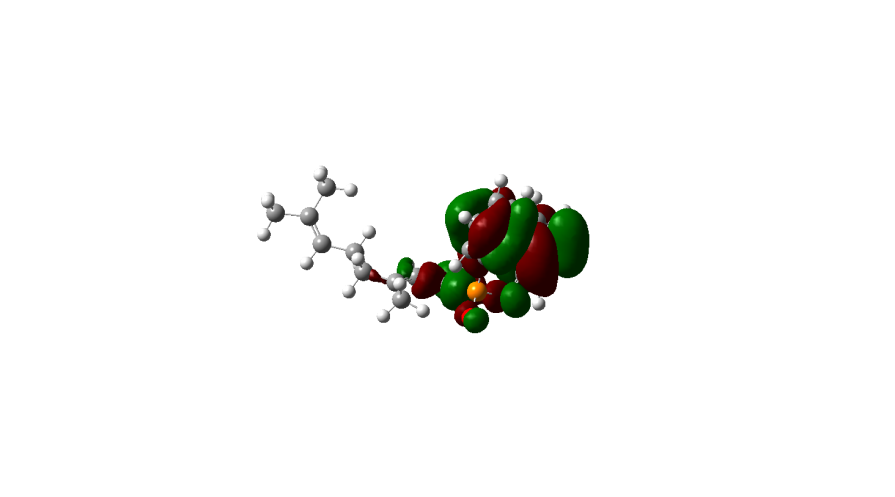 | 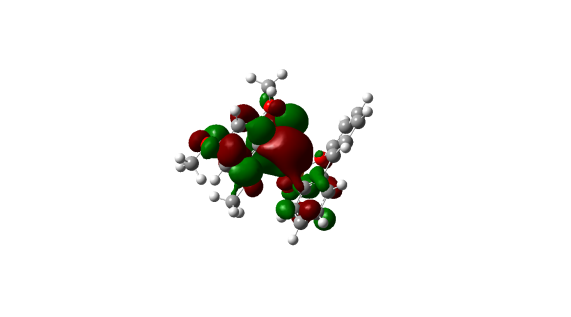  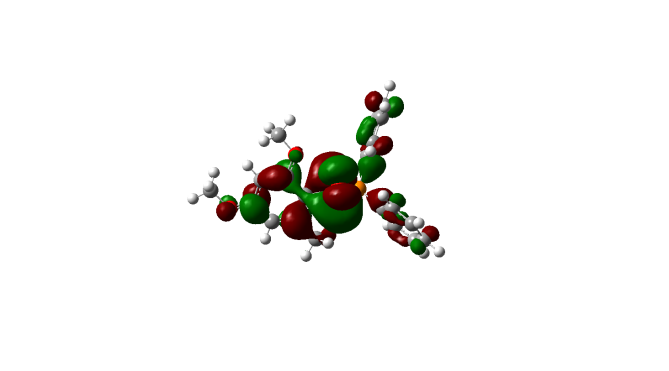  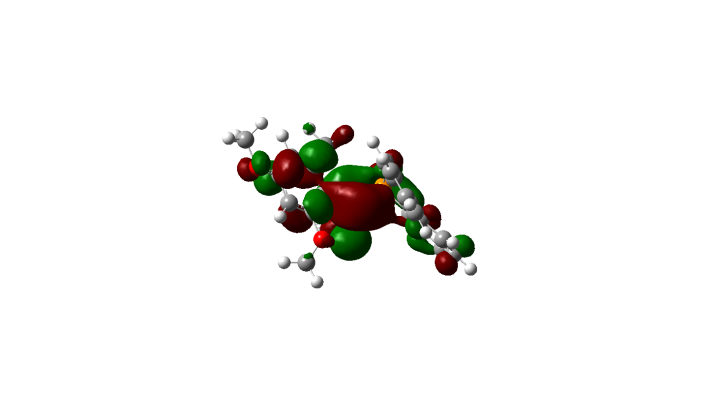  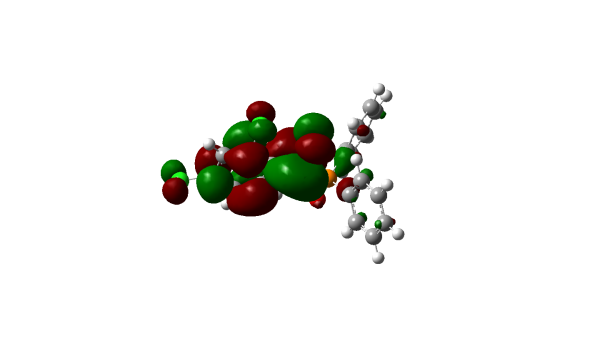  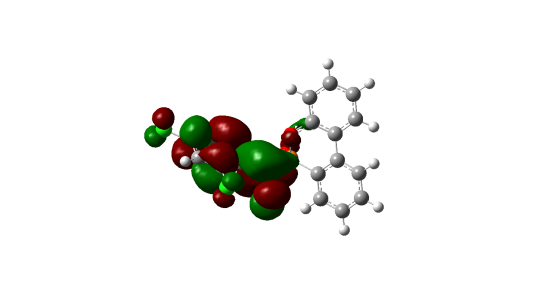  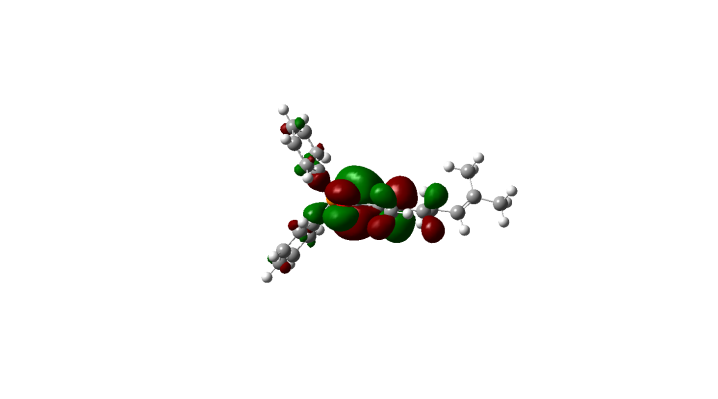  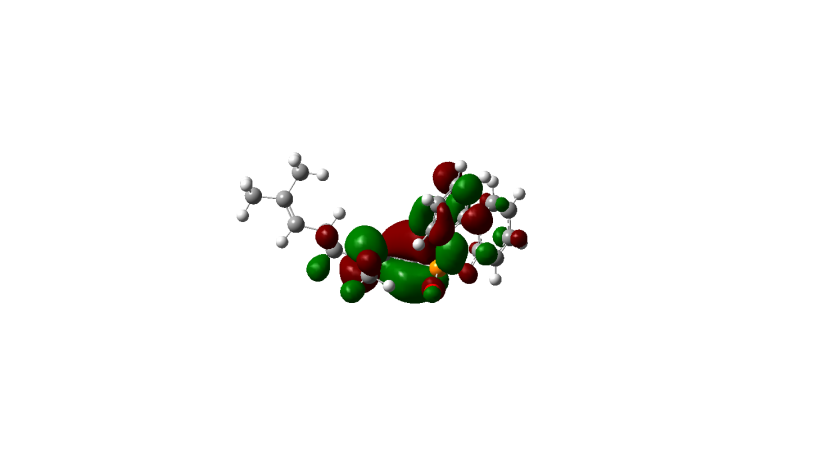 |

**General remarks**

2,4,6-Trimethoxybenzaldehyde (TMO), 2,4-dimethoxy-6-methylbenzaldehyde (DMO), 2,4-dichlorobenzaldehyde (DC), Citral (1:1 mixture of 2E- and 2Z-3,7-dimethyl-2,6-octadienal (geranial and neral respectively)), 9,10-dihydro-9-oxa-10-phosphaphenanthrene 10-oxide (DOPO), diphenylphosphine oxide (DPO), 2,4,6-trimethylbenzoyldiphenylphosphine oxide (TPO), Dess-Martin periodinane (DMP), trimethylolpropane triacrylate (TMPTA), dichloromethane (DCM), ethyl acetate (AcOEt), phenyl-N-*tert*-butylnitrone (PBN), *tert*-butylbenzene were purchased from TCI and were used directly without additional purifications. ^1^H-NMR, and ^31^P-NMR spectra were performed at ambient temperature using a Varian Oxford 300 MHz spectrometer, Bruker-500MHz spectrometer and CDCl_3_ as the solvent, with the chemical shifts in ^1^H-NMR aligned to the solvent peak of CHCl_3_ in the deuterated solvent (7.26 ppm). A Bruker high-resolution mass spectrometer (HRMS) was used to record high-resolution mass spectra.


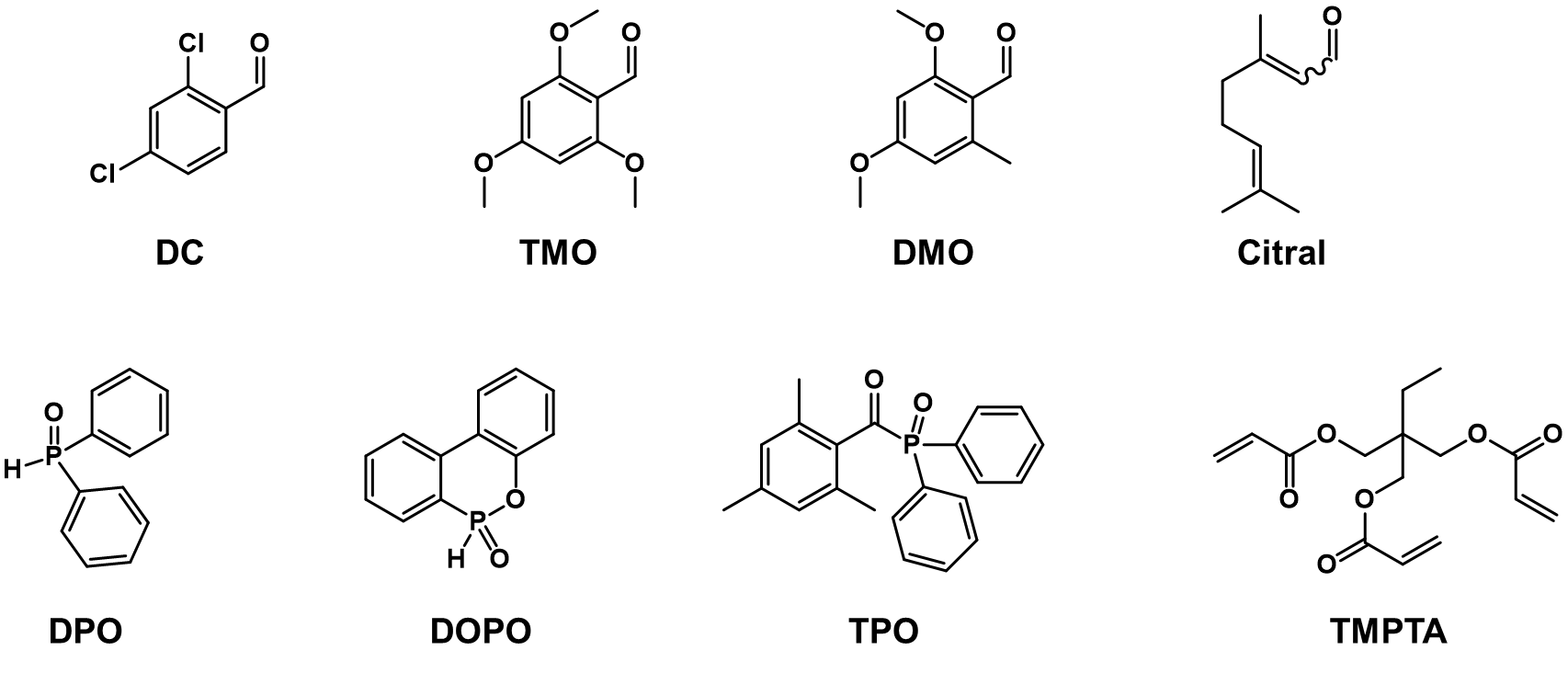


Figure S1. The compounds used in the study.

**Synthesis of DOPO- and DPO-based photoinitiators**

**General procedure:**

Photoinitiators were synthesized via a two-step route. First, DOPO or DPO was reacted with the corresponding aldehyde (2,4,6-trimethoxybenzaldehyde, 2,4-dimethoxy-6-methylbenzaldehyde, 2,4-dichlorobenzaldehyde, or citral) under nucleophilic addition conditions to form the intermediate alcohols (e.g., TMO-DPO-ALC, DMO-DPO-ALC, Citral-DPO-ALC). Subsequently, the alcohols were oxidized using DMP to generate the target acylphosphine oxides or acylphosphinates (TMO-DPO, DMO-DPO, DC-DPO, Citral-DPO, TMO-DOPO, DMO-DOPO, DC-DOPO, Citral-DOPO).

Using Citral as a bio-based starting material:

Citral was reacted with DPO or DOPO to produce Citral-DPO-ALC and Citral-DOPO-ALC intermediates. Oxidation of these intermediates with DMP afforded Citral-DPO and Citral-DOPO.

**Synthesis of (diphenylphosphoryl)(2,4,6-trimethoxyphenyl)methanone (TMO-DPO)**

This compound was synthesized using the previously reported synthetic method [1].

^1^H NMR (500 MHz, CDCl_3_) δ(ppm): δ 7.88 – 7.80 (m, 4H), 7.52 – 7.40 (m, 6H), 6.07 (s, 2H), 3.79 (s, 3H), 3.60 (s, 6H).[1]


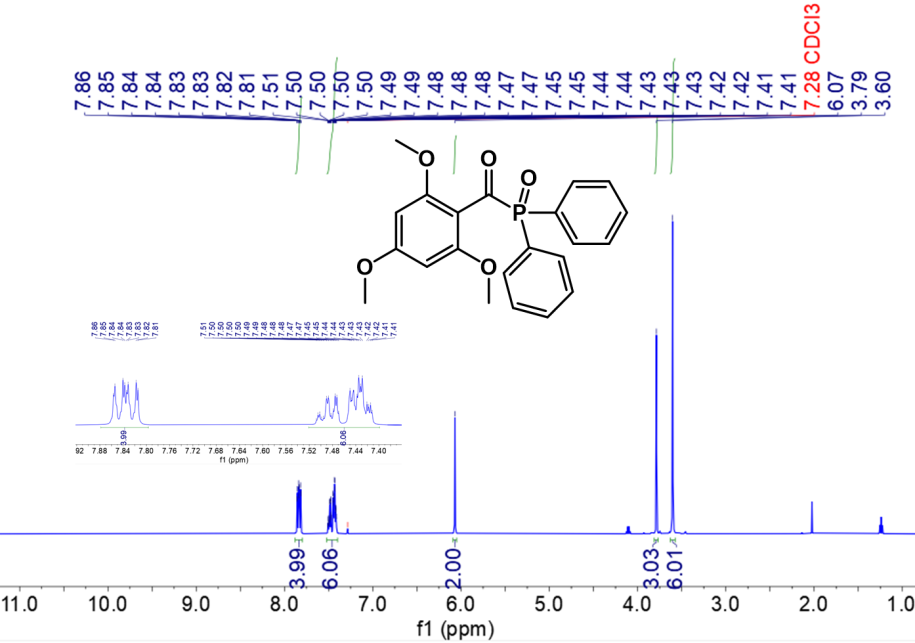


Figure S2. ^1^H-NMR spectrum of TMO-DPO.

**Synthesis of (6-oxidodibenzo[c,e][1,2]oxaphosphinin-6-yl)(2,4,6-trimethoxyphenyl) methanone (TMO-DOPO)**

**Step 1: Synthesis of 6-(hydroxy(2,4,6-trimethoxyphenyl)methyl)dibenzo[c,e][1,2] oxaphosphinine 6-oxide (TMO-DOPO-ALC)**

TMO (1.0 equiv., 1 mmol, 196 mg) and DOPO (1.0 equiv., 1 mmol, 216 mg) were added to AcOEt (10 mL). The reaction mixture was then stirred at room temperature for 24 hours. The mixture obtained by vacuum (0.1 mbar) rotary evaporation of the mixture was separated by column chromatography to obtain TMO-DOPO-ALC and then directly used in the second step (Transparent gels, 317 mg, 77% isolated yield, mixture of two diastereoisomers). ^1^H NMR (500 MHz, CDCl_3_) δ (ppm): δ 7.98 – 7.89 (m, 0.5H), 7.88 – 7.82 (m, 1H), 7.81 – 7.74 (m, 1.5H), 7.67 (t, *J* = 7.8 Hz, 0.3H), 7.62 (t, *J* = 7.7 Hz, 0.7H), 7.48 (td, *J* = 7.5, 1.0 Hz, 0.3H), 7.40 (td, *J* = 7.5, 1.0 Hz, 0.7H)., 7.34 – 7.24 (m, 1H), 7.19 – 7.09 (m, 1.7H), 7.04 (dd, *J* = 8.1, 1.3 Hz, 0.3H), 6.02 (s, 0.5H), 5.85 (s, 1.4H), 5.69 – 5.56 (m, 1H), 4.51 – 4.34 (m, 1H), 3.78 (s, 0.8H), 3.71 (s, 2.2H), 3.57 (s, 1.6H), 3.52 (s, 4.3H).

^31^P NMR (202 MHz, CDCl_3_) δ (ppm): δ 32.0, 30.8.

HRMS (ESI): m/z calculated for [C_22_H_21_NaO_6_P]^+^: 435.0968, found: 435.0972.


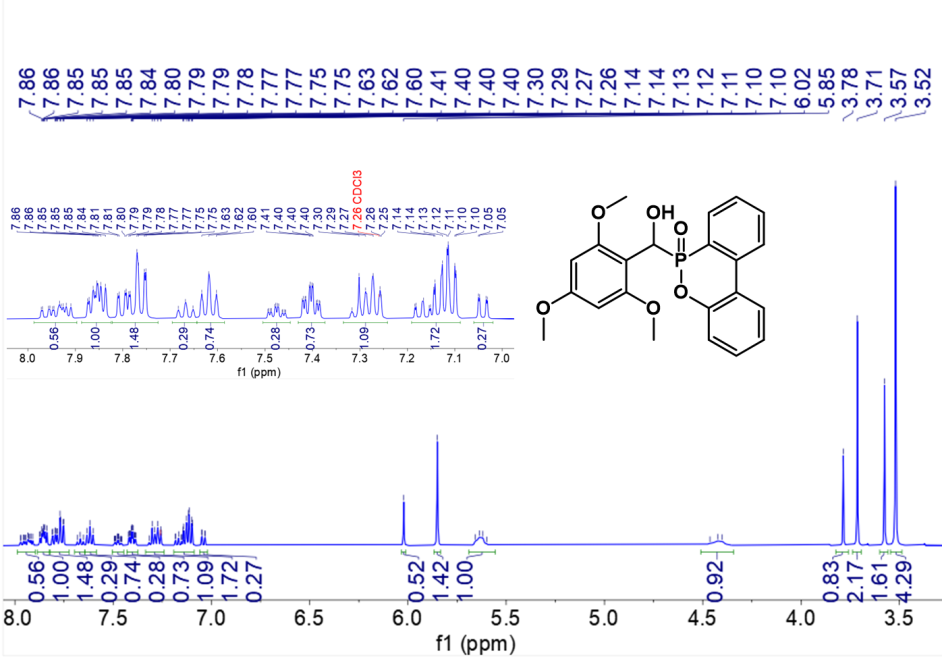


Figure S3. ^1^H-NMR spectrum of TMO-DOPO-ALC.


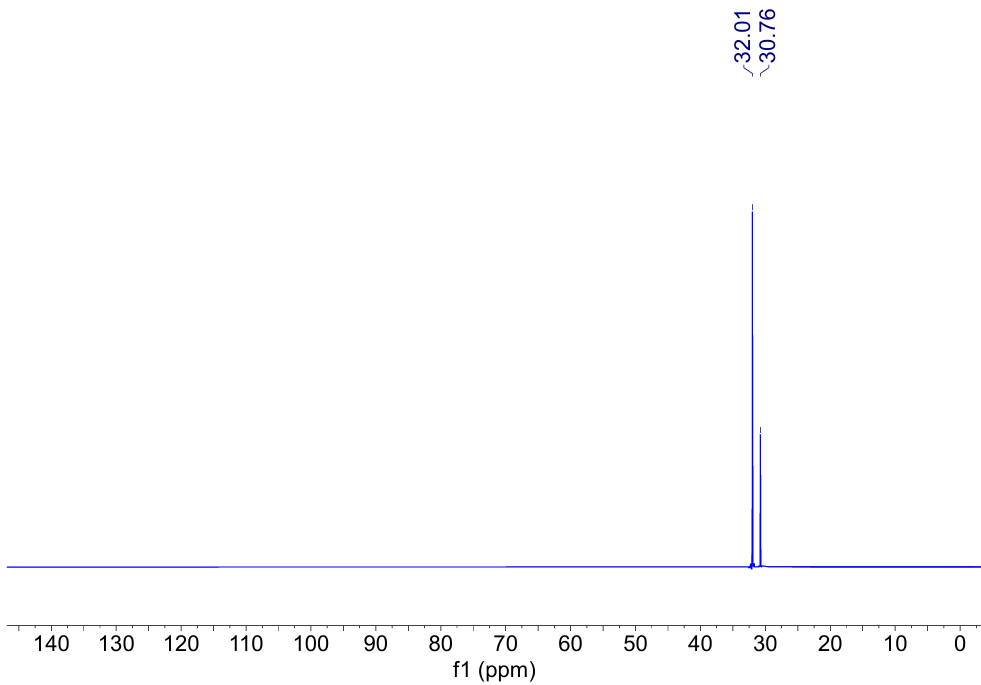


Figure S4. ^31^P-NMR spectrum of TMO-DOPO-ALC.

**Step 2: Synthesis of TMO-DOPO**

Dess-Martin periodinane (1.5 equiv., 3 mmol, 1.27 g) was added to a solution of TMO-DOPO-ALC (1 equiv., 2 mmol, 825 mg) in DCM (2 mL). The reaction mixture was then vigorously stirred in the dark for 4 h and quenched with an aqueous saturated solution of Na_2_SO_3_ (3 mL). The resulting mixture was then extracted with AcOEt (20 mL). The organic phase was washed successively with aqueous saturated solutions of NaHCO_3_ (15 mL) and Na_2_SO_3_ (15 mL). The organic phase was then concentrated under vacuum. A light yellow product (TMO-DOPO) was obtained and no additional purifications were required (714 mg, 87% isolated yield). (NMR spectra in Figures S5).

^1^H NMR (500 MHz, CDCl_3_) δ(ppm): ^1^H NMR (500 MHz, CDCl_3_) δ 7.95 – 7.86 (m, 2H), 7.83 (dd, *J* = 8.0, 1.6 Hz, 1H), 7.65 (t, *J* = 7.8 Hz, 1H), 7.47 (tdd, *J* = 7.5, 3.0, 1.0 Hz, 1H), 7.25 (t, *J* = 7.7 Hz, 1H), 7.16 (td, *J* = 7.6, 1.3 Hz, 1H), 7.05 (dd, *J* = 8.1, 1.3 Hz, 1H), 6.01 (s, 2H), 3.79 (s, 3H), 3.72 (s, 6H).

^31^P NMR (202 MHz, CDCl_3_) δ(ppm): δ 11.2.

HRMS (ESI): m/z calculated for [C_22_H_19_NaO_6_P]^+^: 433.0803, found: 433.0811


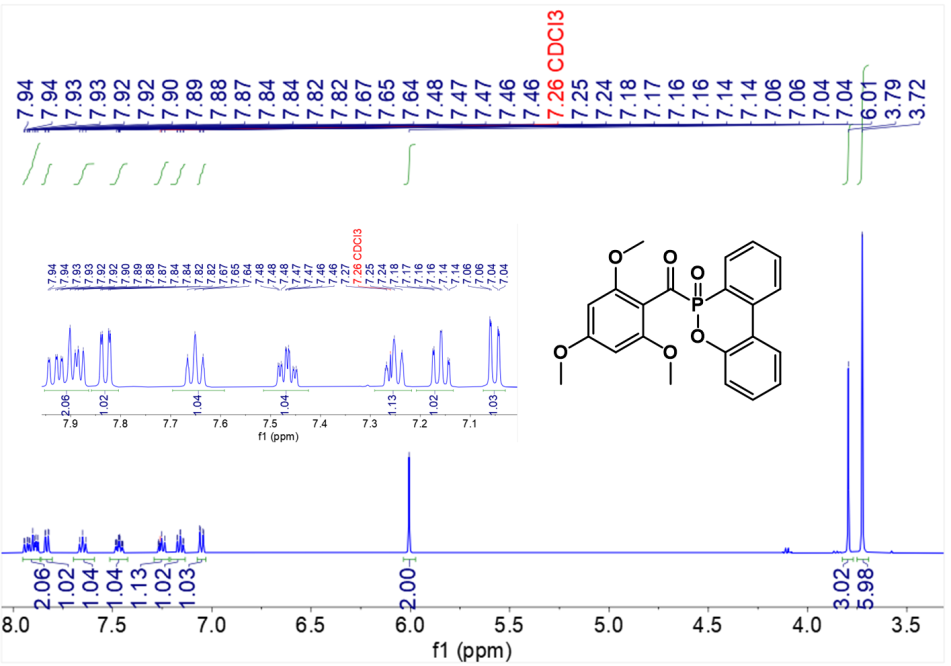


Figure S5. ^1^H-NMR spectrum of TMO-DOPO.


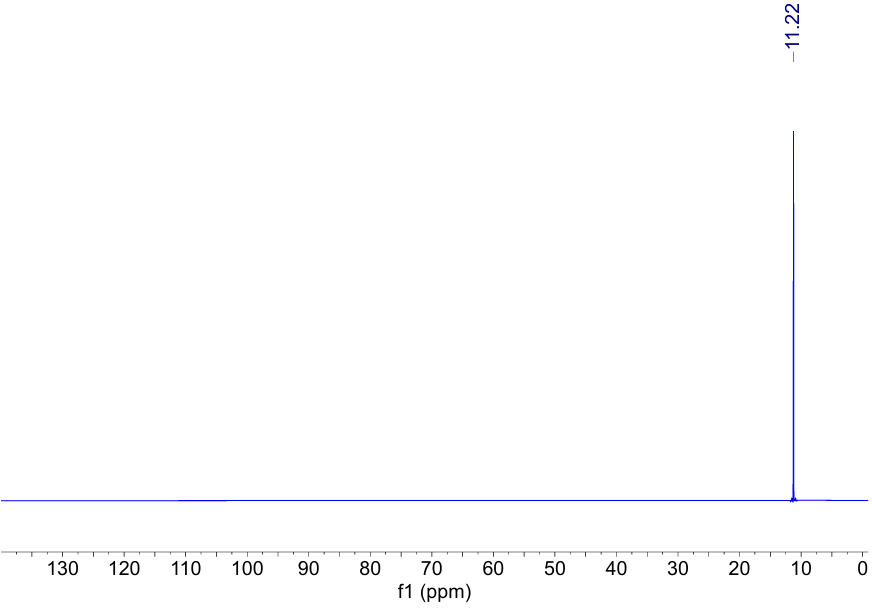


Figure S6. ^31^P-NMR spectrum of TMO-DOPO.

**Synthesis of (2,4-dimethoxy-6-methylphenyl)(diphenylphosphoryl)methanone (DMO-DPO)**

This compound was synthesized using the previously reported synthetic method [1].

**Synthesis of (2,4-dimethoxy-6-methylphenyl)(6-oxidodibenzo[c,e][1,2]oxaphosphinin-6-yl)methanone (DMO**-**DOPO)**

**Step 1:** **Synthesis of 6-((2,4-dimethoxy-6-methylphenyl)(hydroxy)methyl)dibenzo[c,e] [1,2]oxaphosphinine 6-oxide (DMO-DOPO-ALC)**

DMO (1.0 equiv., 1 mmol, 196 mg) and DOPO (1.0 equiv., 1 mmol, 216 mg) were added to AcOEt (10 mL). The reaction mixture was then stirred at room temperature for 24 hours. The mixture obtained by vacuum (0.1 mbar) rotary evaporation of the mixture was separated by column chromatography to obtain DMO-DOPO-ALC and then directly used in the second step (Transparent gels, 316 mg, 80% isolated yield, mixture of two diastereoisomers). ^1^H NMR (500 MHz, CDCl_3_) δ (ppm): δ 8.02 – 7.76 (m, 2.6H), 7.71 – 7.58 (m, 1.4H), 7.50 – 7.27 (m, 1.6H), 7.25 – 7.04 (m, 2.2H), 6.36 (d, *J* = 2.4 Hz, 0.4H), 6.22 – 6.05 (m, 1.6H), 5.51 – 5.36 (m, 1H), 4.58 – 4.27 (m, 1H), 3.78 (s, 1.2H), 3.71 (s, 1.7H), 3.49 (s, 1.7H), 3.26 (s, 1.2H), 2.37 (s, 1.2H), 2.22 (s, 1.7H).

^31^P NMR (202 MHz, CDCl_3_) δ (ppm): δ 32.3, 32.1.

HRMS (ESI): m/z calculated for [C_22_H_21_NaO_5_P]^+^: 419.1019 found: 419.1024.


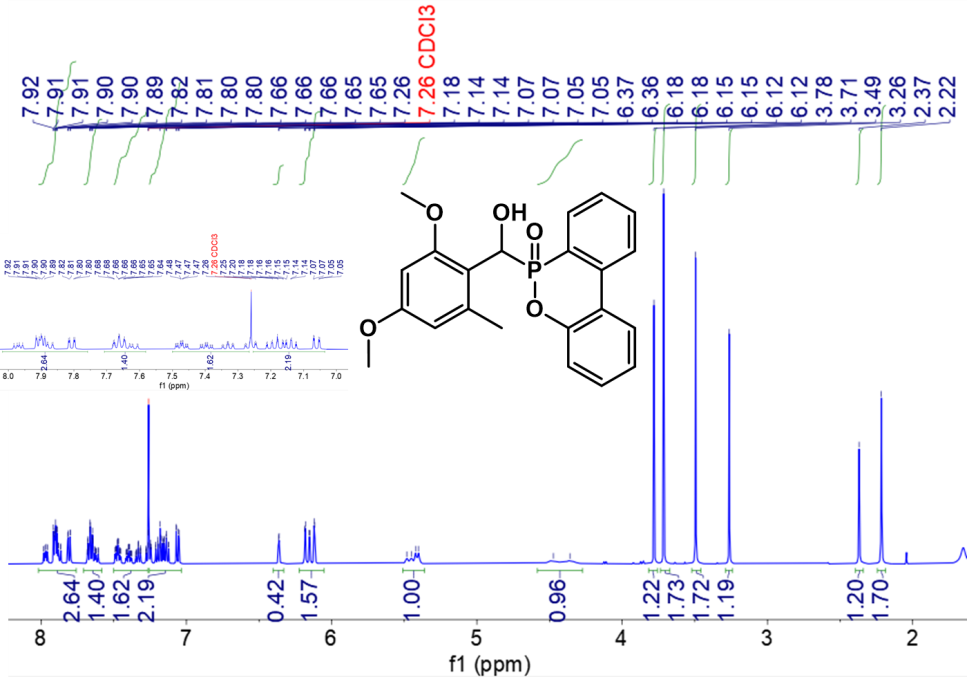


Figure S7. ^1^H-NMR spectrum of DMO-DOPO-ALC.


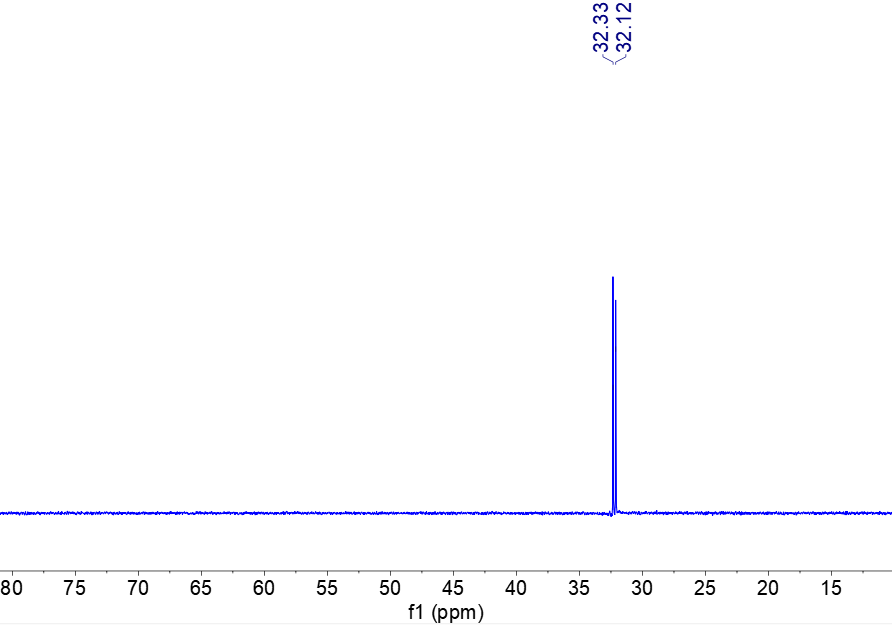


Figure S8. ^31^P-NMR spectrum of DMO-DOPO-ALC.

**Step 2: Synthesis of DMO-DOPO**

Dess-Martin periodinane (1.5 equiv., 3 mmol, 1.27 g) was added to a solution of DMO-DOPO-ALC (1 equiv., 2 mmol, 789 mg) in DCM (2 mL). The reaction mixture was then vigorously stirred in the dark for 4 h and quenched with an aqueous saturated solution of Na_2_SO_3_ (3 mL). The resulting mixture was then extracted with AcOEt (20 mL). The organic phase was washed successively with aqueous saturated solutions of NaHCO_3_ (15 mL) and Na_2_SO_3_ (15 mL). The organic phase was then concentrated under vacuum. A light yellow product (DMO-DOPO) was obtained and no additional purifications were required (709 mg, 90% isolated yield).

^1^H NMR (500 MHz, CDCl_3_) δ(ppm): δ 7.97 – 7.90 (m, 2H), 7.86 (dd, *J* = 7.9, 1.7 Hz, 1H), 7.69 (t, *J* = 7.7 Hz, 1H), 7.51 (tdd, *J* = 7.5, 3.0, 1.0 Hz, 1H), 7.29 (t, *J* = 7.7 Hz, 1H), 7.20 (td, *J* = 7.6, 1.3 Hz, 1H), 7.08 (dd, *J* = 8.1, 1.4 Hz, 1H), 6.30 – 6.23 (m, 2H), 3.88 (s, 3H), 3.81 (s, 3H), 2.02 (s, 3H).

^31^P NMR (202 MHz, CDCl_3_) δ(ppm): δ 11.4.


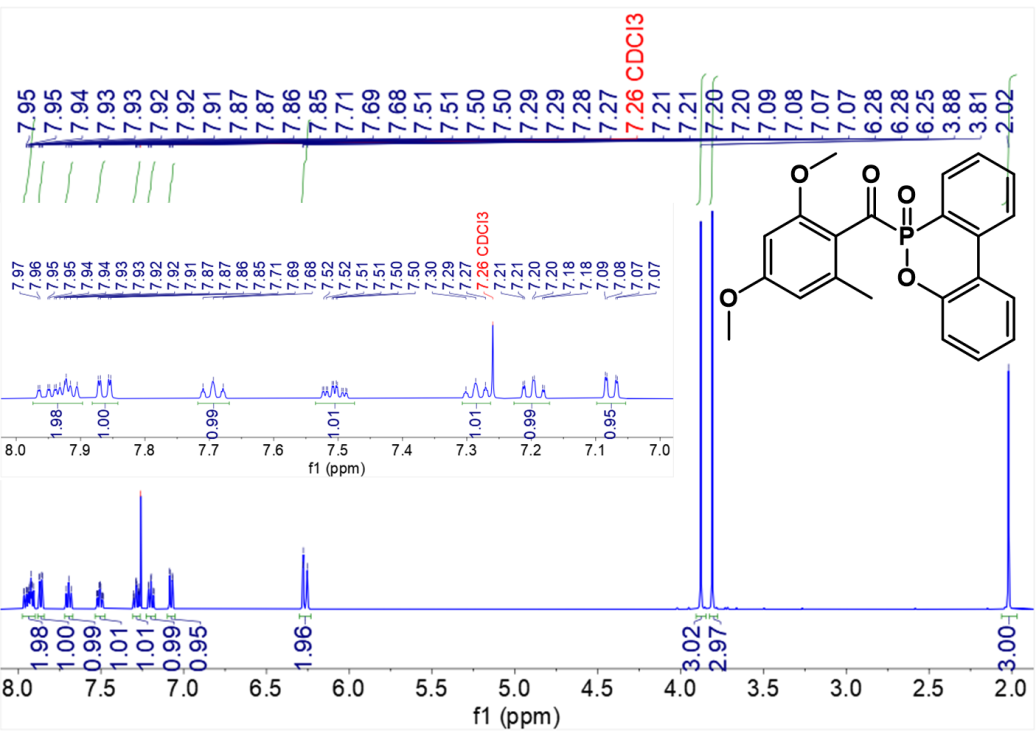


Figure S9. ^1^H-NMR spectrum of DMO-DOPO.


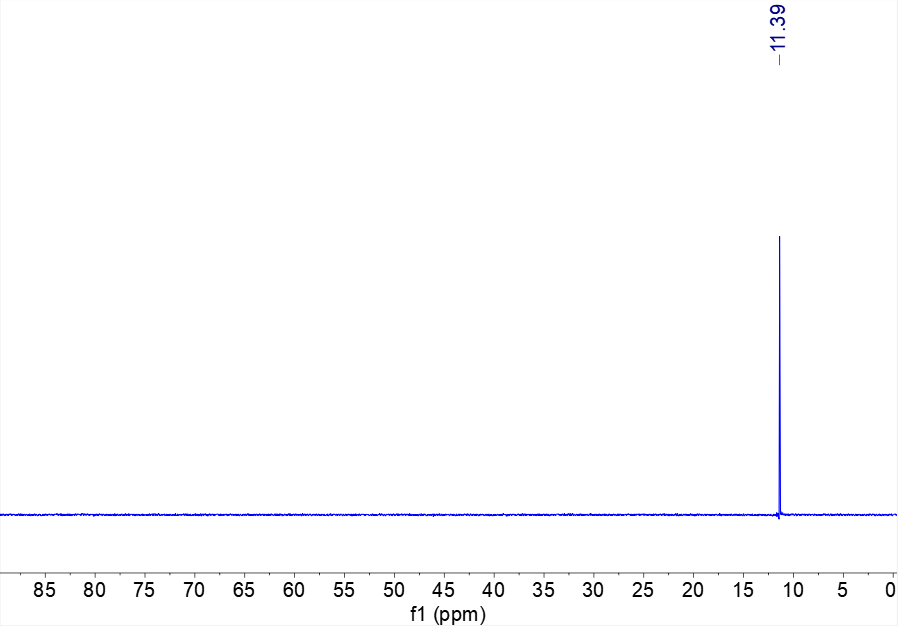


Figure S10. ^31^P-NMR spectrum of DMO-DOPO.

**Synthesis of (2,4-dichlorophenyl)(diphenylphosphoryl)methanone (DC-DPO)**

**Step 1: Synthesis of ((2,4-dichlorophenyl)(hydroxy)methyl)diphenylphosphine oxide (DC-DPO-ALC)**

DC (1.0 equiv., 1 mmol, 196 mg) and DPO (1.0 equiv., 1 mmol, 202 mg) were added to AcOEt (10 mL). The reaction mixture was then stirred at room temperature for 24 hours. After the reaction, the mixture was filtered through a sand core funnel to afford a white solid. This solid was then washed with AcOEt (3×25 mL) and dried under vacuum (0.1 mbar). DC-DPO-ALC was obtained (358 mg; 90 % yield). DC-DPO-ALC was then directly used in the second step without additional purifications.

**Step 2:** **Synthesis of DC-DPO**

Dess-Martin periodinane (1.5 equiv., 3 mmol, 1.27 g) was added to a solution of DC-DPO-ALC (1 equiv., 2 mmol, 782 mg) in DCM (2 mL). The reaction mixture was then vigorously stirred in the dark for 4 h and quenched with an aqueous saturated solution of Na_2_SO_3_ (3 mL). The resulting mixture was then extracted with AcOEt (20 mL). The organic phase was washed successively with aqueous saturated solutions of NaHCO_3_ (15 mL) and Na_2_SO_3_ (15 mL). The organic phase was then concentrated under vacuum. A yellow product (DC-DPO) was obtained and no additional purifications were required (593 mg, 79% isolated yield). ^1^H NMR (500 MHz, CDCl_3_) δ (ppm): δ 8.45 (d, *J* = 8.5 Hz, 1H), 7.94 – 7.84 (m, 4H), 7.65 – 7.57 (m, 2H), 7.56 – 7.50 (m, 4H), 7.47 (dd, *J* = 2.1, 1.1 Hz, 1H), 7.37 (dd, *J* = 8.4, 2.0 Hz, 1H).

^31^P NMR (202 MHz, CDCl_3_) δ (ppm): δ 21.7.


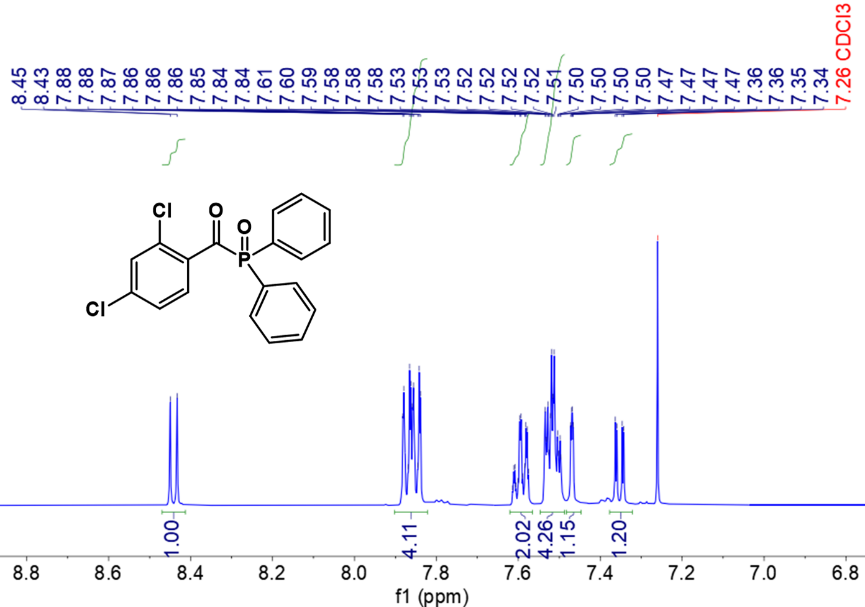


Figure S11. ^1^H-NMR spectrum of DC-DPO.


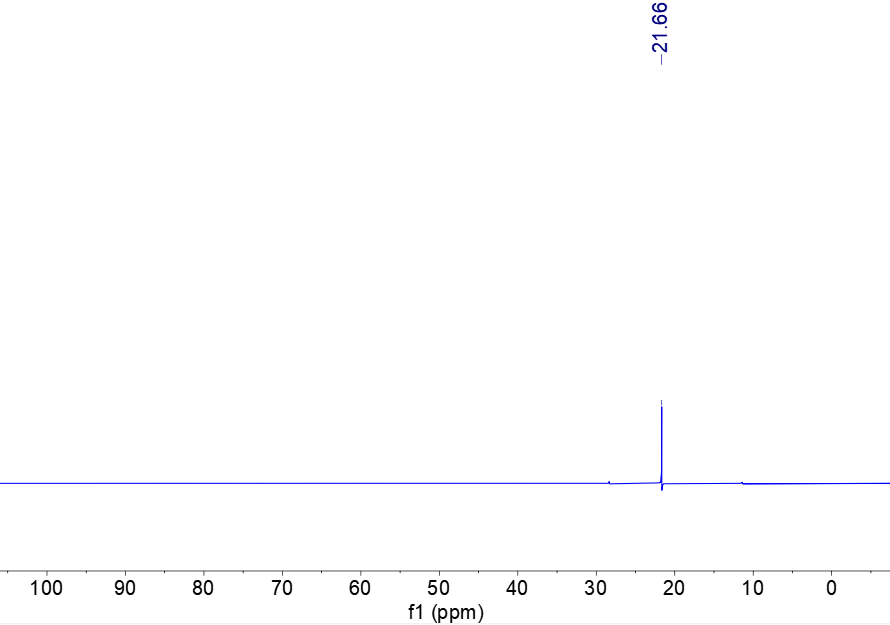


Figure S12. ^31^P-NMR spectrum of DC-DPO.

**Synthesis of (2,4-dichlorophenyl)(6-oxidodibenzo[c,e][1,2]oxaphosphinin-6-yl)methanone (DC-DOPO)**

**Step 1:** **Synthesis of 6-((2,4-dichlorophenyl)(hydroxy)methyl)dibenzo[c,e] [1,2]oxaphosphinine 6-oxide (DC-DOPO-ALC)**

DC (1.0 equiv., 1 mmol, 196 mg) and DOPO (1.0 equiv., 1 mmol, 216 mg) were added to AcOEt (10 mL). The reaction mixture was then stirred at room temperature for 24 hours. After the reaction, the mixture was filtered through a sand core funnel to afford a white solid. This solid was then washed with AcOEt (3×25 mL) and dried under vacuum (0.1 mbar). DC-DOPO-ALC was obtained and then directly used in the second step without additional purifications (white powder, 348 mg, 86% isolated yield, mixture of two diastereoisomers).

^1^H NMR (500 MHz, CDCl_3_) δ (ppm): δ 7.99 (dd, *J* = 11.9, 7.5 Hz, 1H), 7.84 – 7.76 (m, 1H), 7.71 (t, *J* = 7.7 Hz, 1H), 7.61 (d, *J* = 7.9 Hz, 1H), 7.52 (td, *J* = 7.6, 3.0 Hz, 1H), 7.30 – 7.26 (m, 1H), 7.18 (d, *J* = 8.1 Hz, 1H), 7.10 (t, *J* = 7.5 Hz, 1H), 6.98 – 6.84 (m, 3H), 5.84 (d, *J* = 12.0 Hz, 1H).

^31^P NMR (202 MHz, CDCl_3_) δ (ppm): δ 32.4, 29.2.

HRMS (ESI): m/z calculated for [C_19_H_13_Cl_2_NaO_3_P]^+^: 412.9877, found: 412.9868.


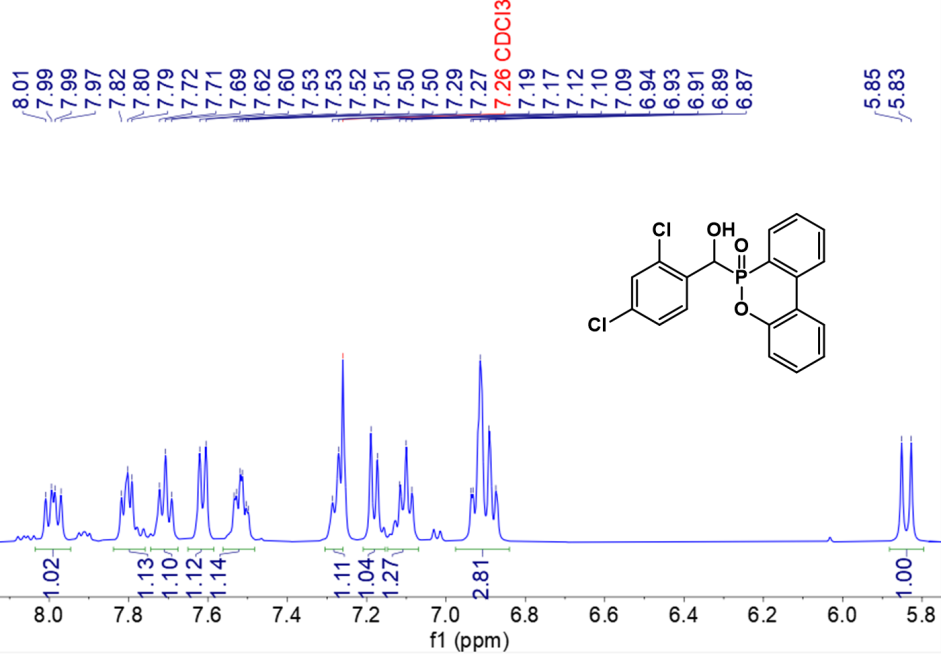


Figure S13. ^1^H-NMR spectrum of DC-DOPO-ALC.


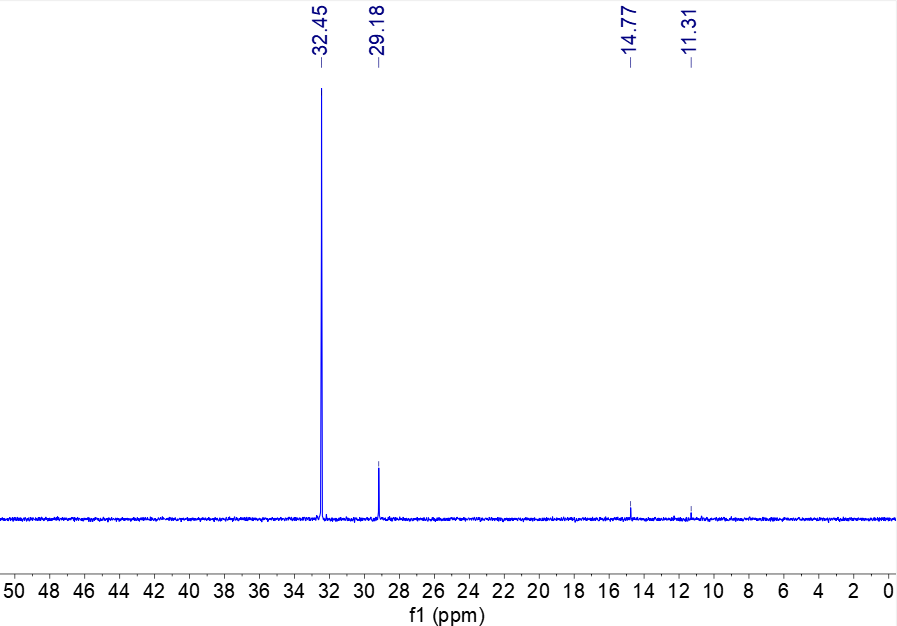


Figure S14. ^31^P-NMR spectrum of DC-DOPO-ALC.

**Step 2:** **Synthesis of DC-DOPO**

Dess-Martin periodinane (1.5 equiv., 3 mmol, 1.27 g) was added to a solution of DC-DOPO-ALC (1 equiv., 2 mmol, 782 mg) in DCM (2 mL). The reaction mixture was then vigorously stirred in the dark for 4 h and quenched with an aqueous saturated solution of Na_2_SO_3_ (3 mL). The resulting mixture was then extracted with AcOEt (20 mL). The organic phase was washed successively with aqueous saturated solutions of NaHCO_3_ (15 mL) and Na_2_SO_3_ (15 mL). The organic phase was then concentrated under vacuum. A yellow product (DC-DOPO) was obtained and no additional purifications were required (701 mg, 89% isolated yield).

^1^H NMR (500 MHz, CDCl_3_) δ (ppm): 8.50 (d, *J* = 8.9 Hz, 1H), 8.04 – 7.94 (m, 2H), 7.93 (dd, *J* = 7.9, 1.7 Hz, 1H), 7.79 (ddt, *J* = 8.4, 7.5, 1.3 Hz, 1H), 7.57 (tdd, *J* = 7.5, 3.1, 1.0 Hz, 1H), 7.49 – 7.44 (m, 2H), 7.34 (td, *J* = 7.3, 1.1 Hz, 1H), 7.29 – 7.26 (m, 1H), 7.12 (dd, *J* = 8.1, 1.3 Hz, 1H).

^31^P NMR (202 MHz, CDCl_3_) δ (ppm): δ 15.2.


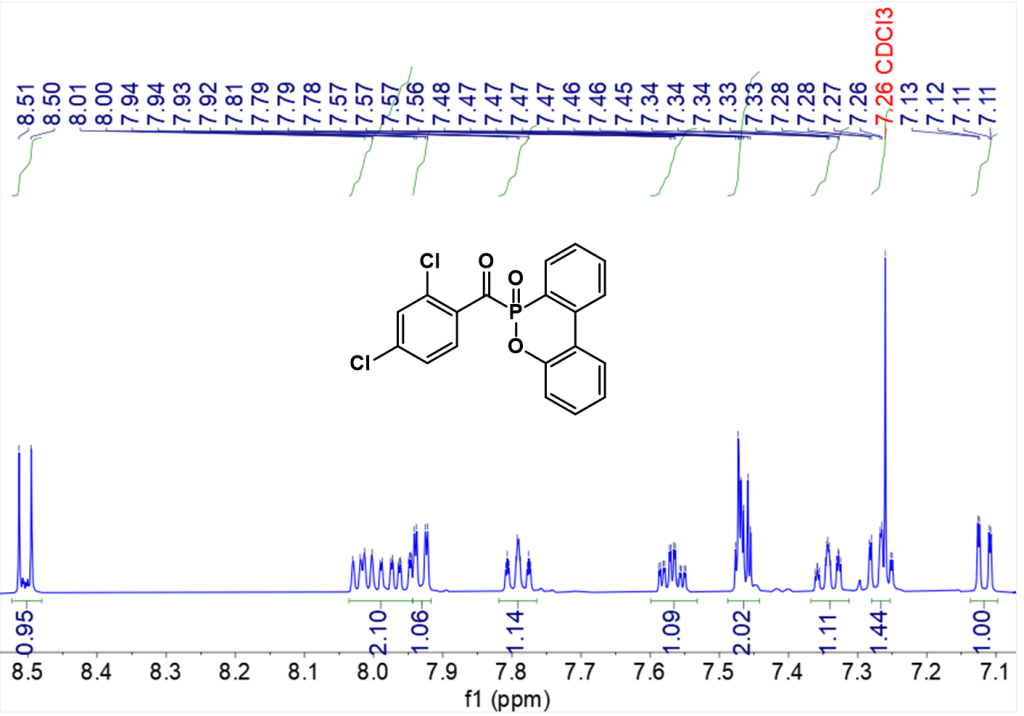


Figure S15. ^1^H-NMR spectrum of DC-DOPO.


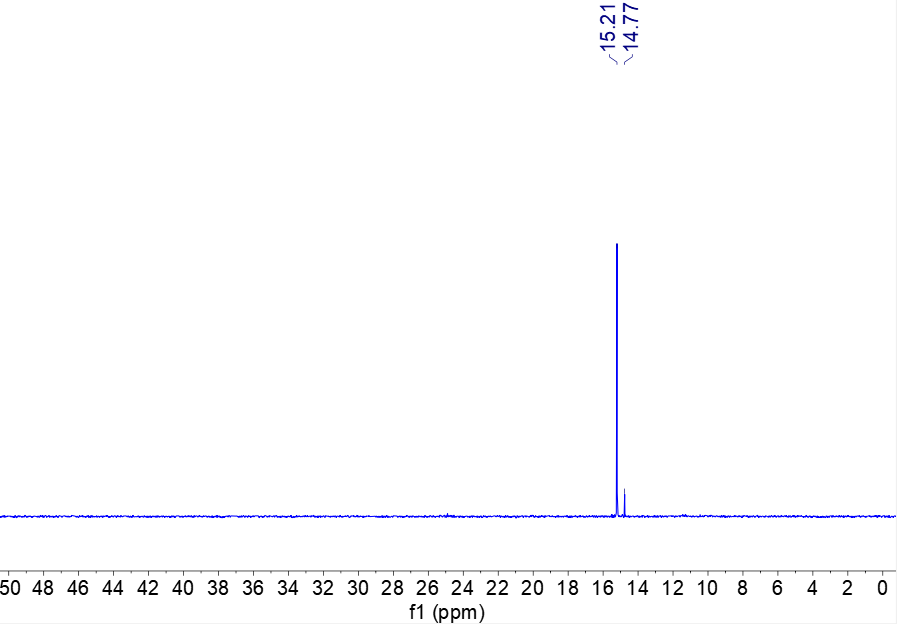


Figure S16. ^31^P-NMR spectrum of DC-DOPO.

**Synthesis of 1-(diphenylphosphoryl)-3,7-dimethylocta-2,6-dien-1-one (Citral-DPO)**

**Step 1:** **Synthesis of (1-hydroxy-3,7-dimethylocta-2,6-dien-1-yl)diphenylphosphine oxide (Citral-DPO-ALC)**

Citral (commercial 1:1 mixture of neral and geranial, 1.0 equiv., 1 mmol, 196 mg) and DPO (1.0 equiv., 1 mmol, 202 mg) were added to AcOEt (10 mL). The reaction mixture was then stirred at room temperature for 24 hours. After the reaction, the mixture was filtered through a sand core funnel to afford a white solid. This solid was then washed with AcOEt (3×25 mL) and dried under vacuum (0.1 mbar). Citral-DPO-ALC was obtained as a mixture of stereo-isomers (white powder, 273 mg, 77% isolated yield, mixture of four stereoisomers).

^1^H NMR (300 MHz, CDCl_3_) δ (ppm): δ 7.96 – 7.85 (m, 2H), 7.83 – 7.71 (m, 2H), 7.57 – 7.40 (m, 6H), 5.42 – 5.24 (m, 1H), 5.10 (d, *J* = 10.0 Hz, 1H), 4.99 (d, *J* = 7.9 Hz, 1H), 2.04 – 1.82 (m, 4H), 1.69 (s, 1.3H), 1.65 (s, 3H), 1.55 (s, 3H), 1.43 (s, 1.3H).

^31^P NMR (121 MHz, CDCl_3_) δ (ppm): δ 31.2, 31.3.

HRMS (ESI): m/z calculated for [C_22_H_27_NaO_2_P]^+^: 377.1641, found: 377.1642.


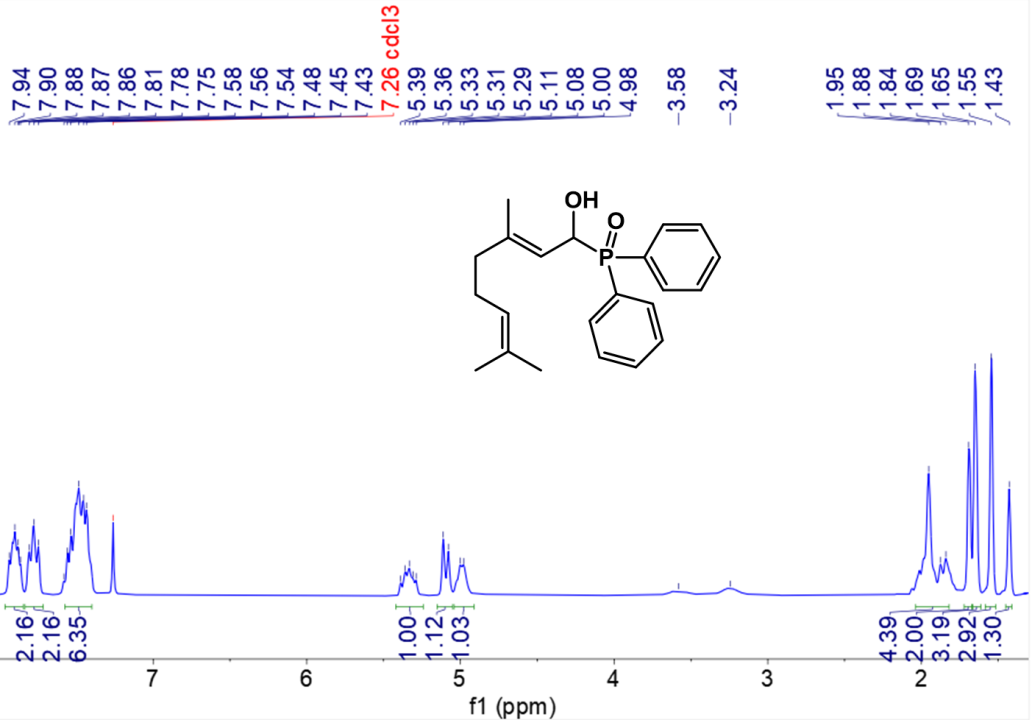


Figure S17. ^1^H-NMR spectrum of Citral-DPO-ALC.


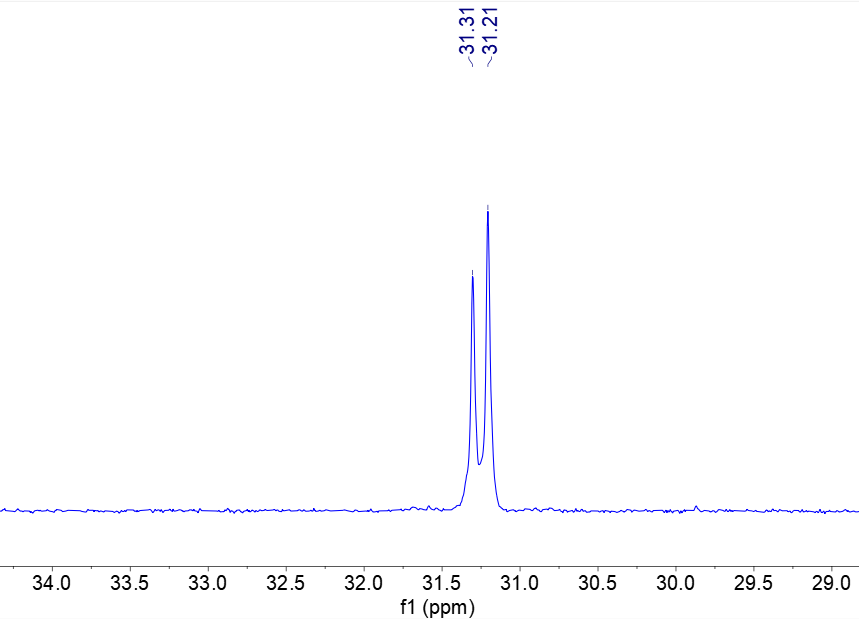


Figure S18. ^31^P-NMR spectrum of Citral-DPO-ALC.

**Step 2:** **Synthesis of Citral-DPO**

Dess-Martin periodinane (1.5 equiv., 3 mmol, 1.27 g) was added to a solution of Citral-DPO-ALC (1 equiv., 2 mmol, 709 mg) in DCM (2 mL). The reaction mixture was then vigorously stirred in the dark for 4 h and quenched with an aqueous saturated solution of Na_2_SO_3_ (3 mL). The resulting mixture was then extracted with AcOEt (20 mL). The organic phase was washed successively with aqueous saturated solutions of NaHCO_3_ (15 mL) and Na_2_SO_3_ (15 mL). The organic phase was then concentrated under vacuum. A yellow product (Citral-DPO) was obtained as a mixture of E and Z diastero-isomers and no additional purifications were required (606 mg, 86% isolated yield).

^1^H NMR (300 MHz, CDCl_3_) δ (ppm): δ 7.87 – 7.77 (m, 4H), 7.56 – 7.43 (m, 6H), 7.19 (s, 1H), 5.16 – 4.88 (m, 1H), 2.64 (t, *J* = 7.7 Hz, 1H), 2.34 – 2.07 (m, 4.4H), 1.98 (s, 1.6H), 1.62 (s, 3H), 1.55 (s, 3H).

^31^P NMR (121 MHz, CDCl_3_) δ (ppm): δ 16.8, 16.9.


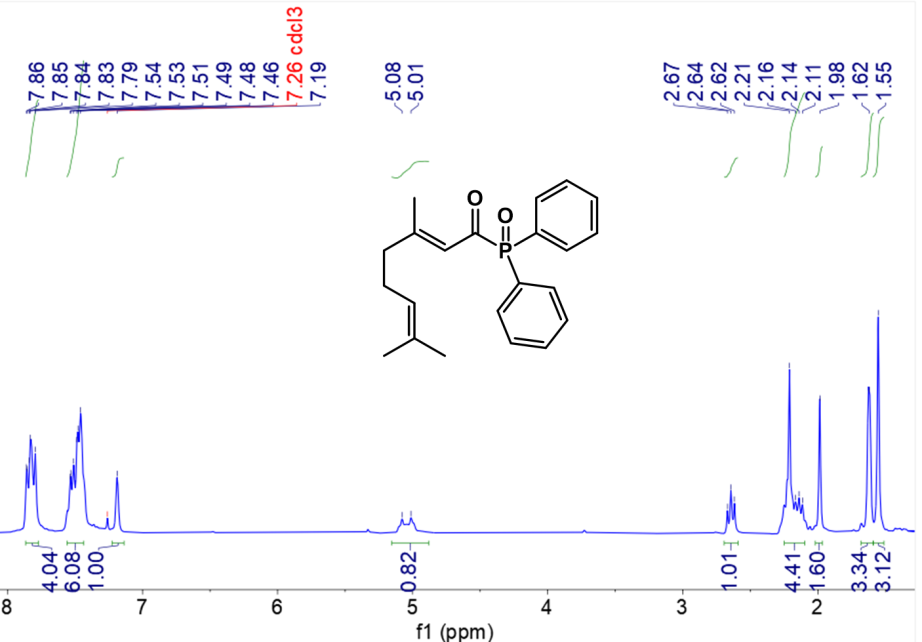


Figure S19. ^1^H-NMR spectrum of Citral-DPO.


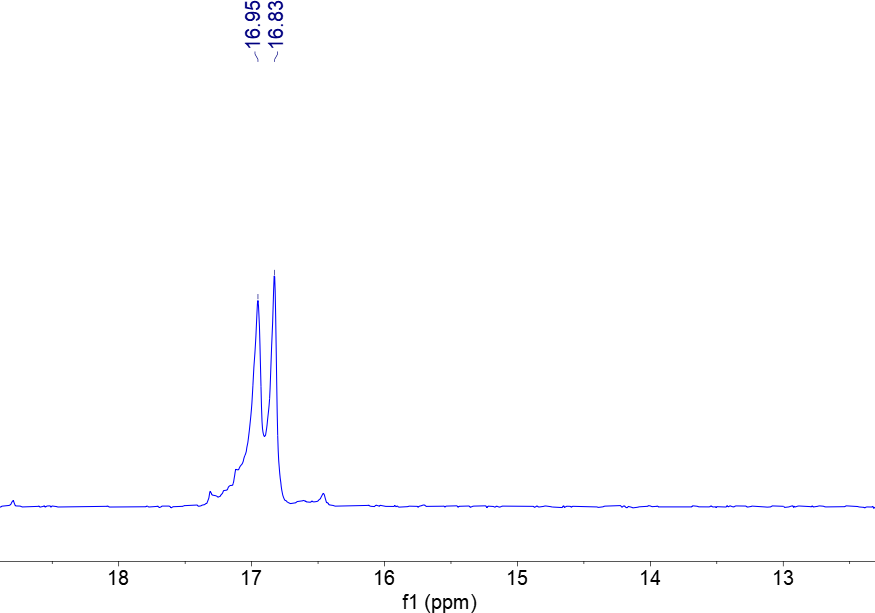


Figure S20. ^31^P-NMR spectrum of Citral-DPO.

**Synthesis of the 3,7-dimethyl-1-(6-oxidodibenzo[c,e][1,2]oxaphosphinin-6-yl)octa-2,6-dien-1-one (Citral-DOPO)**

**Step 1: Synthesis of 6-(1-hydroxy-3,7-dimethylocta-2,6-dien-1-yl)dibenzo[c,e][1,2] oxaphosphinine 6-oxide (Citral-DOPO-ALC)**

Citral (commercial 1:1 mixture of neral and geranial, 1.0 equiv., 1 mmol, 196 mg) and DOPO (1.0 equiv., 1 mmol, 216 mg) were added to AcOEt (10 mL). The reaction mixture was then stirred at room temperature for 24 hours. After the reaction, the mixture was filtered through a sand core funnel to afford a white solid. This solid was then washed with AcOEt (3×25 mL) and dried under vacuum (0.1 mbar). Citral-DOPO-ALC was obtained as a mixture of stereo-isomers (white powder, 280 mg, 76% isolated yield, mixture of four diastereoisomers).

^1^H NMR (300 MHz, CDCl_3_) δ (ppm): δ 8.11 – 7.99 (m, 1H), 7.98 – 7.91 (m, 1H) 7.68 (t, *J* = 7.8 Hz, 1H), 7.51 – 7.45 (m, 1H), 7.34 – 7.27 (m, 1H), 7.22 – 7.12 (m, 2H), 5.14 – 4.65 (m, 2.6H), 2.03 – 1.72 (m, 2.8H), 1.64 (s, 3.4H), 1.57 (s, 2H), 1.55 – 1.51 (m, 1.4H), 1.45 (s, 3H), 1.33 (s, 0.8H).

^31^P NMR (121 MHz, CDCl_3_) δ (ppm): δ 32.8, 32.5, 31.6, 31.1.

HRMS (ESI): m/z calculated for [C_22_H_25_NaO_3_P]^+^: 391.1436, found: 391.1434.


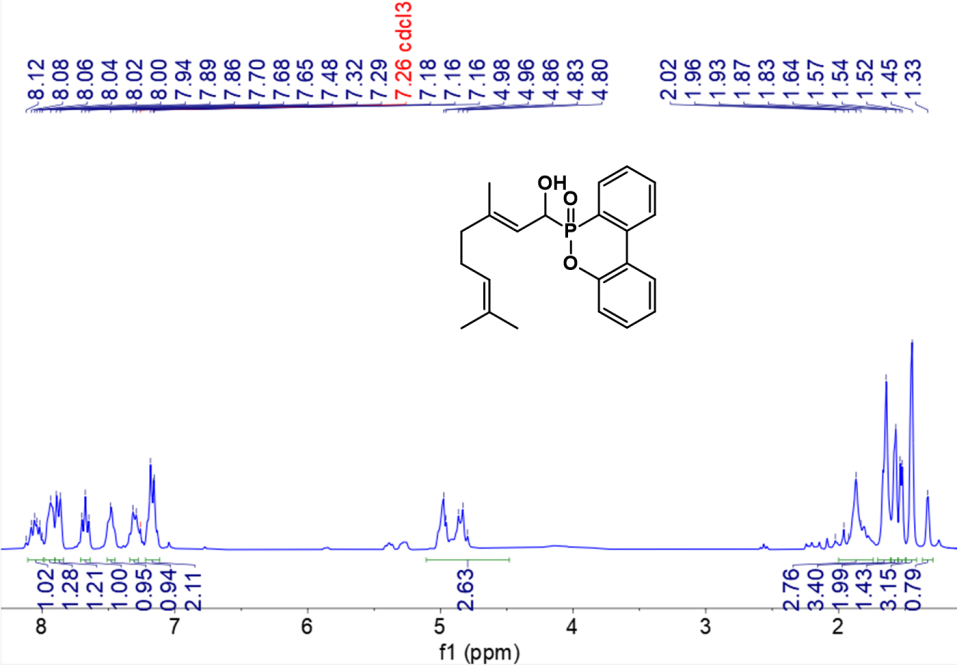


Figure S21. ^1^H-NMR spectrum of Citral-DOPO-ALC.


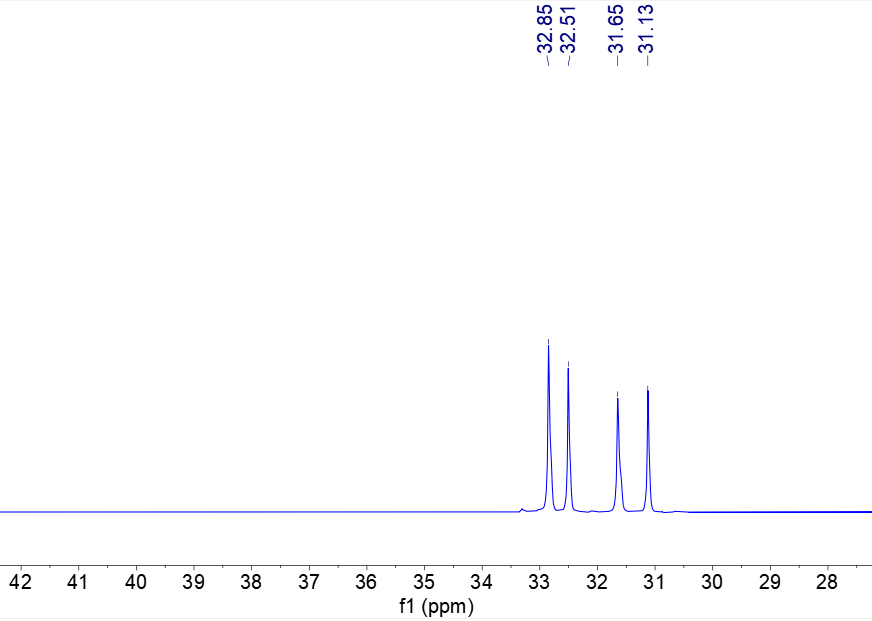


Figure S22. ^31^P-NMR spectrum of Citral-DOPO-ALC.

**Step 2:** **Synthesis of Citral-DOPO**

Dess-Martin periodinane (1.5 equiv., 3 mmol, 1.27 g) was added to a solution of Citral-DOPO-ALC (1 equiv., 2 mmol, 737 mg) in DCM (2 mL). The reaction mixture was then vigorously stirred in the dark for 4 h and quenched with an aqueous saturated solution of Na_2_SO_3_ (3 mL). The resulting mixture was then extracted with AcOEt (20 mL). The organic phase was washed successively with aqueous saturated solutions of NaHCO_3_ (15 mL) and Na_2_SO_3_ (15 mL). The organic phase was then concentrated under vacuum A yellow product (Citral-DPO) was obtained as a mixture of E and Z diastero-isomers and no additional purifications were required (660 mg, 90% isolated yield). It was shown that this product is not stable during storage (see below).

^1^H NMR (300 MHz, CDCl_3_) δ (ppm): δ 8.02 – 7.96 (m, 1H), 7.94 – 7.86 (m, 2H), 7.75 – 7.69 (m, 1H), 7.50 (td, *J* = 7.5, 3.1 Hz, 1H), 7.35 (t, *J* = 7.7 Hz, 1H), 7.21 (d, *J* = 8.1 Hz, 2H), 6.95 (s, 1H), 5.11 – 4.79 (m, 1H), 2.23 (d, *J* = 11.7 Hz, 2H), 2.12 (s, 3H), 1.68 (s, 3H), 1.60 (s, 3H), 1.51 (s, 1.3H), 1.45 (s, 0.7H).

^31^P NMR (121 MHz, CDCl_3_) δ (ppm): δ 12.8, 12.9.


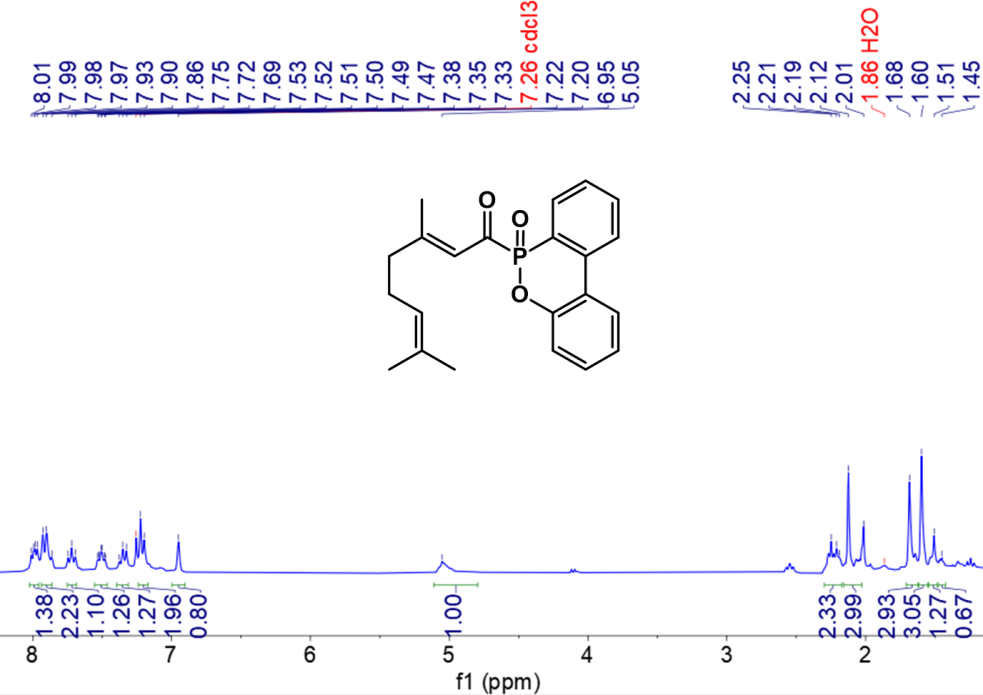


Figure S23. ^1^H-NMR spectrum of Citral-DOPO.


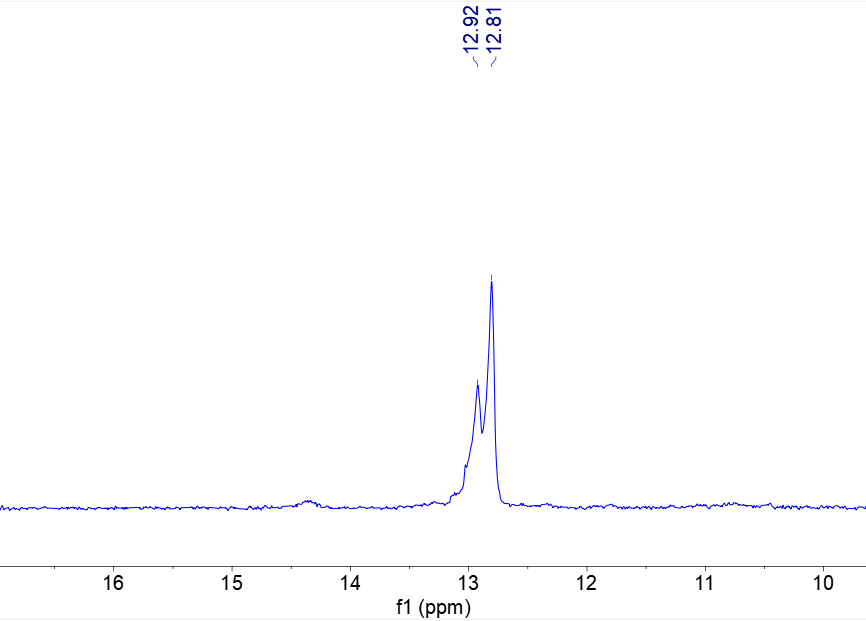


Figure S24. ^31^P-NMR spectrum of Citral-DOPO.

**Storage stability of the synthesized photoinitiators given in Figure S25**

Figures S26, S27, 28, 29, 30, 31 show the ^1^H NMR spectra of the prepared TMO-DOPO, DMO-DOPO, DC-DPO, DC-DOPO, Citral-DPO and Citral-DOPO after it was sealed in the dark for 2 weeks. TMO-DOPO, DMO-DOPO were stable. DC-DPO, DC-DOPO, Citral-DPO and Citral-DOPO were not stable.


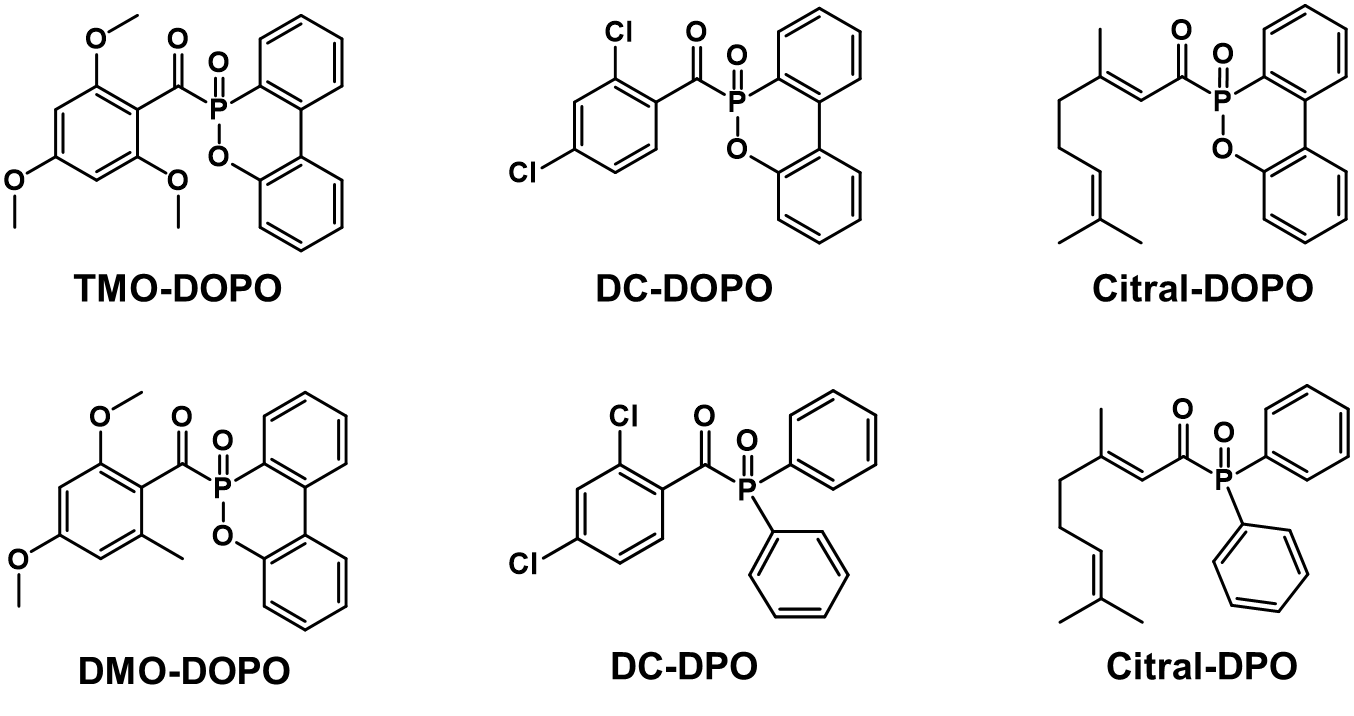


Figure S25. Molecules for Stability Testing.


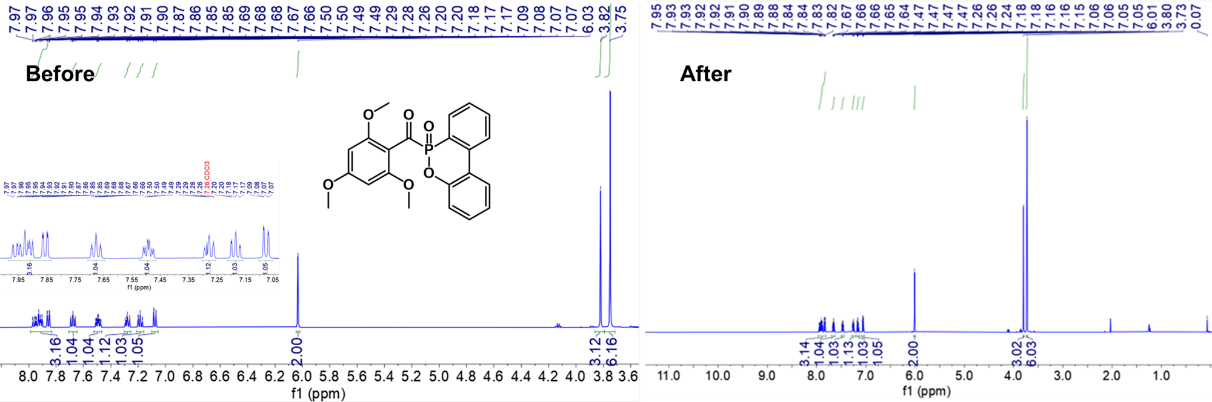


Figure S26. ^1^H-NMR spectrum of TMO-DOPO.


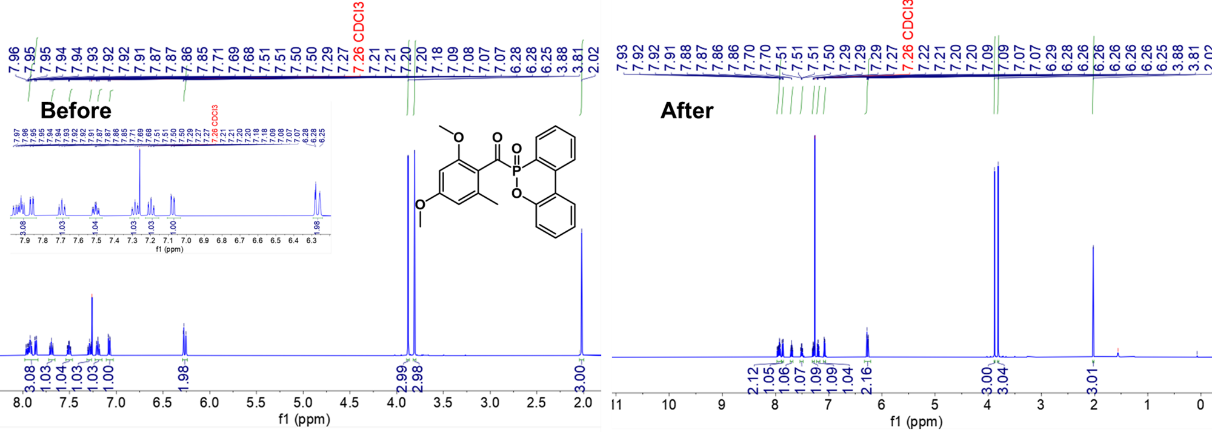


Figure S27. ^1^H-NMR spectrum of DMO-DOPO.


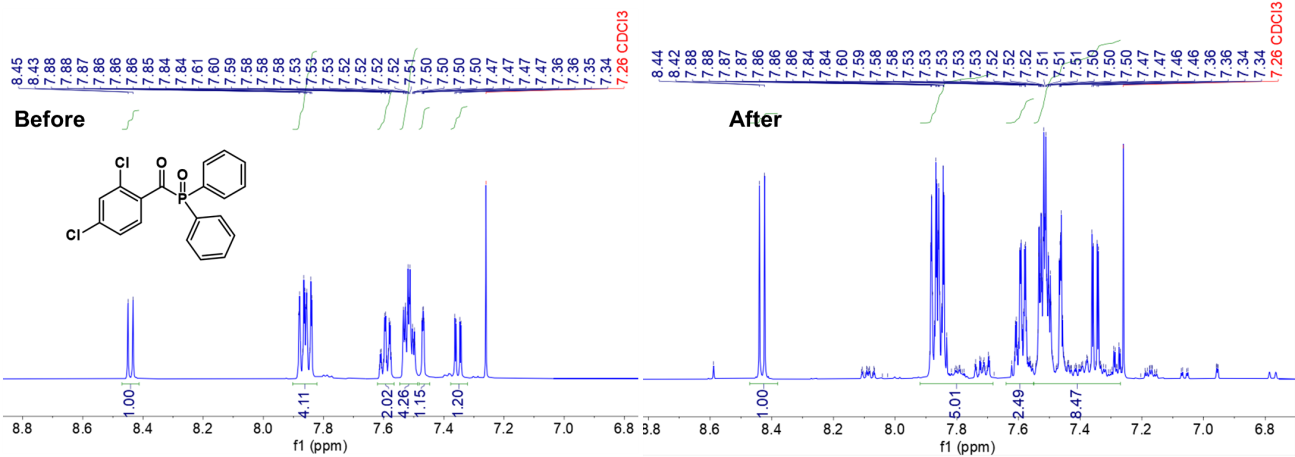


Figure S28. ^1^H-NMR spectrum of DC-DPO.


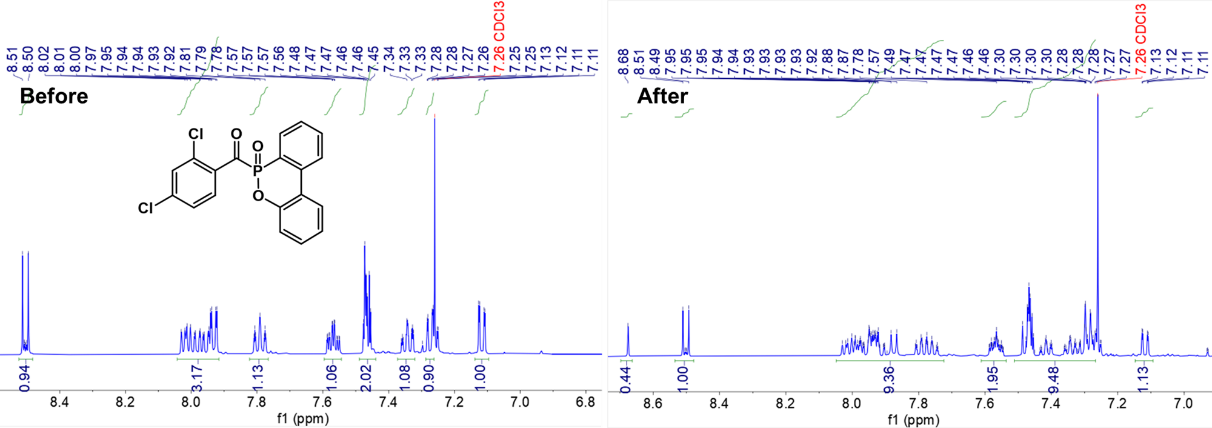


Figure S29. ^1^H-NMR spectrum of DC-DOPO.


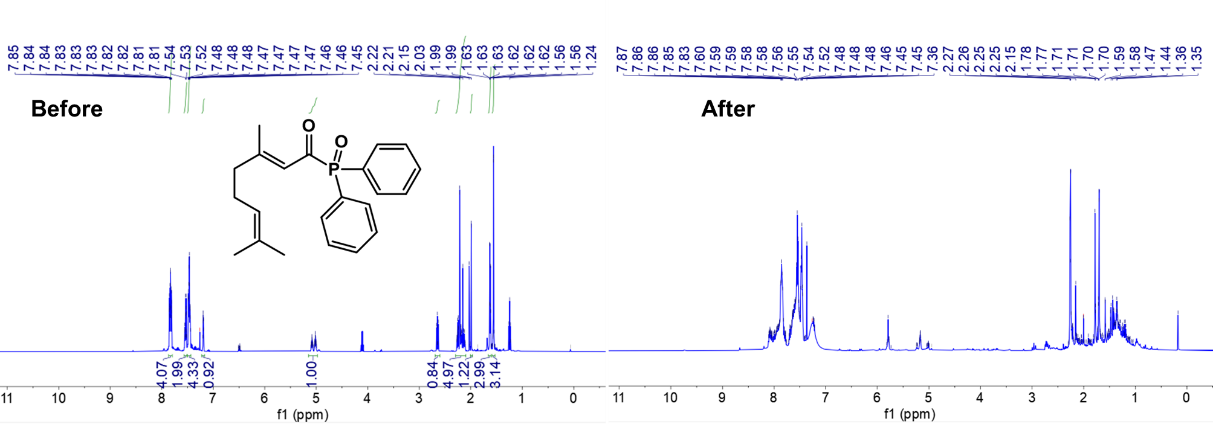


Figure S30. ^1^H-NMR spectrum of Citral-DPO.


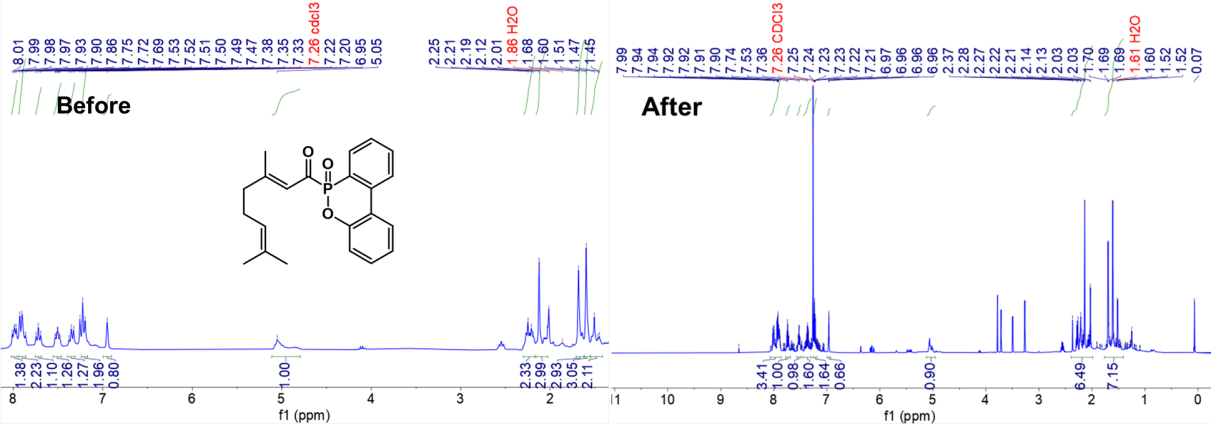


Figure S31. ^1^H-NMR spectrum of Citral-DOPO.

**UV-visible spectroscopy experiments**

The ultraviolet-visible spectra were performed using a Jasco V-730 spectrometer in a quartz cell with DCM as the solvent. In experiments involving steady-state photolysis, a photoinitiator solution in DCM underwent irradiation using LED@385 nm (100 mW/cm^2^), UV-vis spectra were recorded at different irradiation times to monitor the process.

**Quantum yield of photolysis**

The quantum yield (Φ) of photolysis was gauged using constant irradiation in a MeCN solution. The quantum yield of photolysis for TPO was quantified according to the value reported in reference [1] (The value of Φ_TPO_ was 0.56). The subsequent formula computes the quantum yield of photolysis for the novel photoinitiator:

Φ_sample_ = Φ_TPO_[ε_TPO_·slope_sample_]/[ε_sample_·slope_TPO_] (Equation 1)

where the slope represents the slope of the function ln[exp(2.3OD_385 nm_ at different irradiation time) - 1] = f(t) (OD: the optical density or absorption).


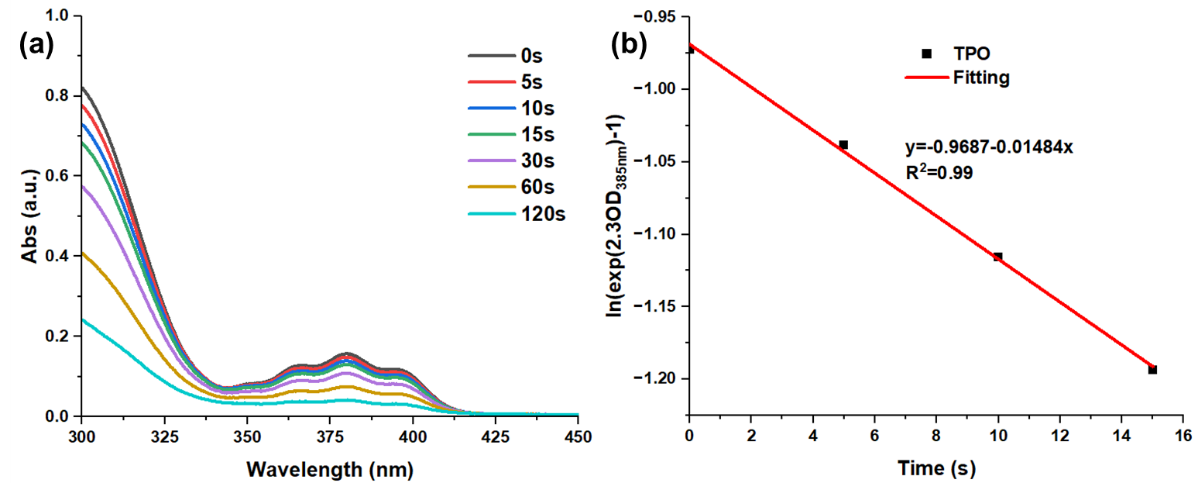


Figure S32. (a) UV-vis absorption spectra recorded during steady-state photolysis of the TPO under LED@385 nm irradiation. (b) The slope of the function.


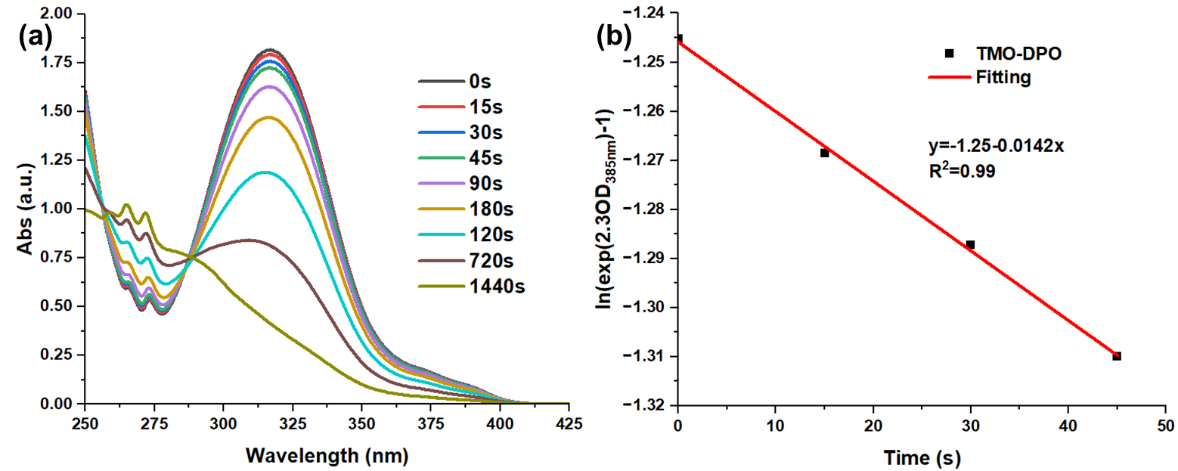


Figure S33. (a) UV-vis absorption spectra recorded during steady-state photolysis of the TMO-DPO under LED@385 nm irradiation. (b) The slope of the function.


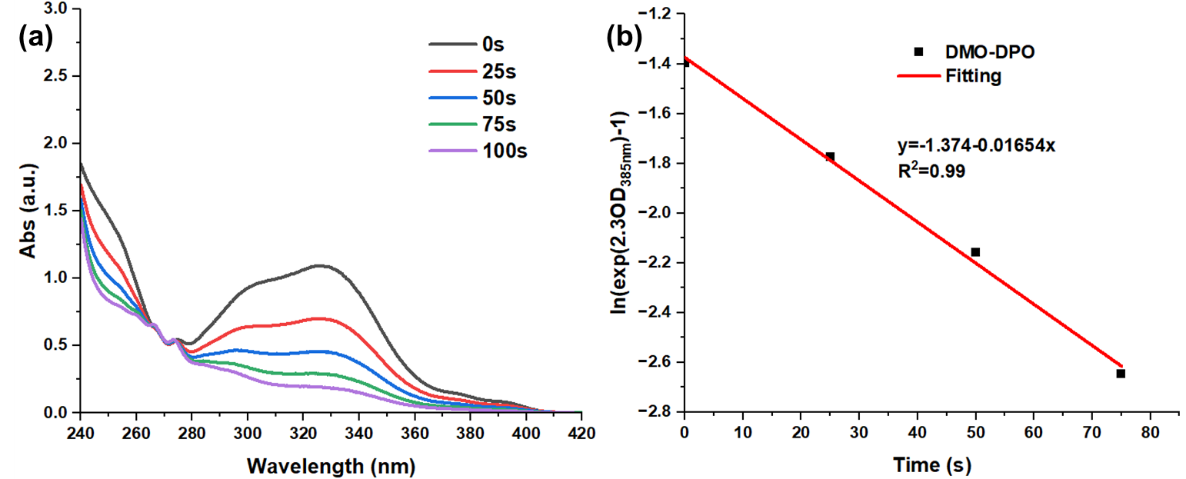


Figure S34. (a) UV-vis absorption spectra recorded during steady-state photolysis of the DMO-DPO under LED@385 nm irradiation. (b) The slope of the function.


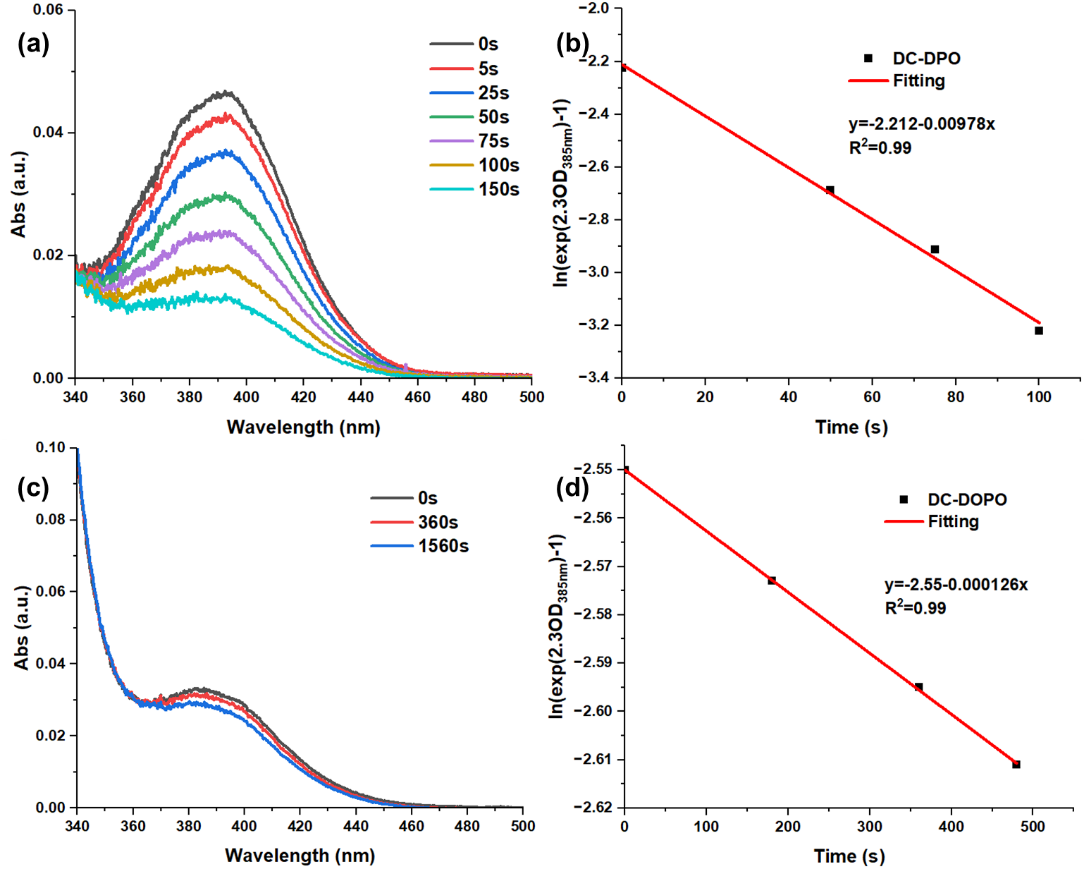


Figure S35. UV-vis absorption spectra recorded during steady-state photolysis of (a) DC-DPO and (c) DC-DOPO under LED@385 nm irradiation. The slope of the function:(b) DC-DPO and (d) DC-DOPO


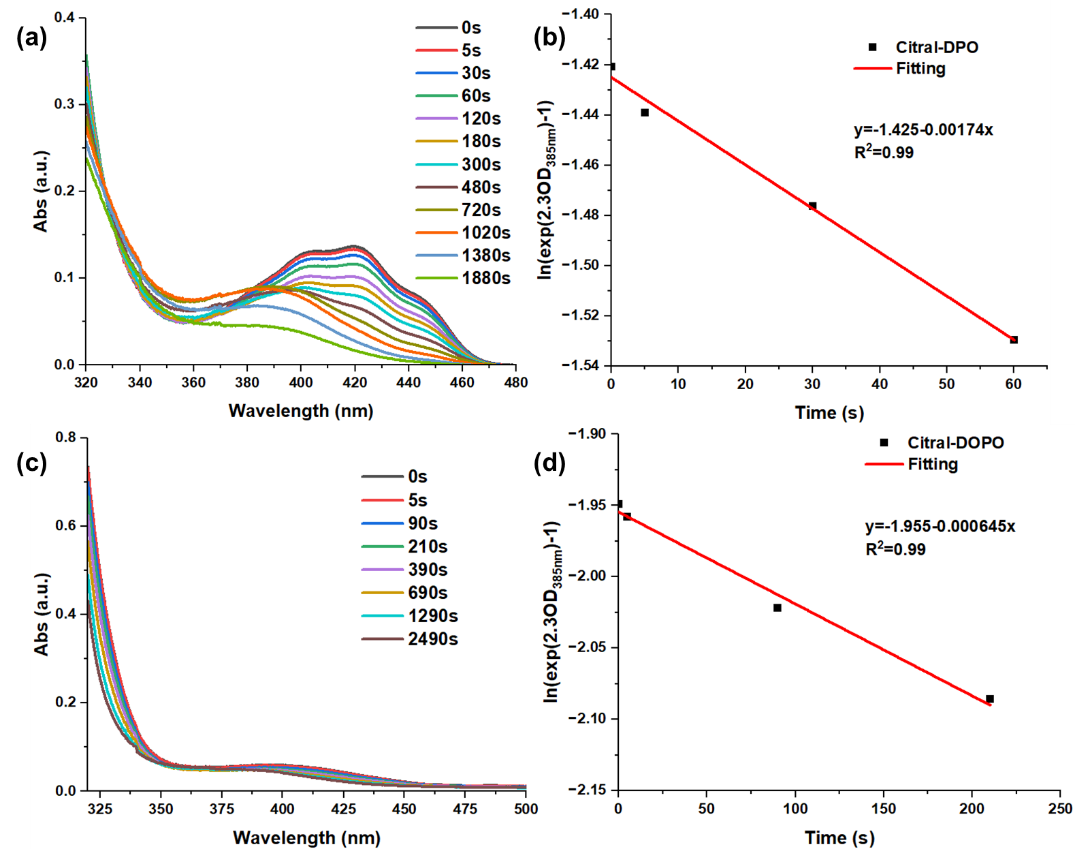


Figure S36. UV-vis absorption spectra recorded during steady-state photolysis of (a) Citral-DPO and (c) Citral-DOPO under LED@385 nm irradiation. The slope of the function:(b) Citral-DPO and (d) Citral-DOPO.

**RT-FTIR spectroscopic analysis**

To obtain photocurable samples, 2.80×10^-5^mol·g^-1^ photoinitiator was added straightly to the trifunctional acrylate monomer trimethylolpropane triacrylate (TMPTA) and stirred overnight until it was dissolved. To track the C=C double bond conversion over time during the photopolymerization of a sample measuring 2 mm or 25μm in thickness, a Jasco 6600 real-time Fourier transform infrared spectrometer (RT-FTIR) was employed. Each photopolymerization test was conducted in air at ambient temperature. The decrease of the acrylate C=C double bond peak is monitored at approximately 1630 cm^-1^. In the photopolymerization tests, a 405 nm LED emitting 100 mW/cm^2^ of light was employed to cure the photosensitive resins. To calculate the double bond conversions over time (DCt), the following formula was used:

DC_t_ = [(A_0_ - A_t_)/ A_0_]×100%

where A_0_ represents the acrylate functional group's peak area at 0 seconds, and At denotes the acrylate functional group's peak area at t seconds.


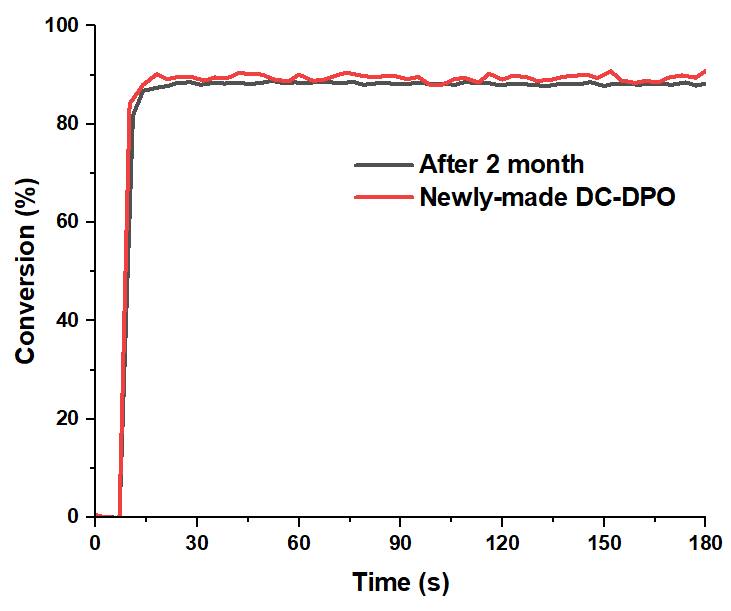


Figure S37. Photopolymerization kinetics of TMPTA with photoinitiators (2.80×10^-5^mol·g^-1^_TMPTA_) under 405 nm LED irradiation: in a thick sample (∼2 mm). Irradiation commenced at t = 10 s.

**DOPO as a hydrogen donor**

Building on the above results, attention was directed to the intrinsic chemical reactivity of the P–H bond in the DOPO structure (structure showed in Figure S1), which may influence its photochemical behavior. Consequently, EPR experiments were carried out on unmodified DOPO to investigate its radical formation characteristics. (Figure S38a, S38c). The results showed that the hyperfine coupling parameters were consistent with those of the oxygen-centered radicals generated by DOPO, with a_N_ = 13.5 G and a_H_ = 1.8 G, indicating that the three materials can spontaneously generate the same type of radicals under similar conditions. Therefore, it is speculated that DOPO and its derivatives may also exhibit hydrogen donor behavior under light irradiation conditions. Combining ITX (isopropylthioxanthone) as a typical Type II photoinitiator, its initiation mechanism typically involves the hydrogen transfer process between the triplet excited state ITX (³ITX) and hydrogen donor molecules. Therefore, in this study, it is necessary to further investigate whether DOPO can serve as an effective hydrogen donor in this system. Due to the presence of active P–H bonds in the structure of DOPO, the following reaction pathway may occur after ITX excitation:

^3^ITX^∗^+DOPO-H→ITX-H∙+DOPO∙

Figure S38b and S38d shows that multiple types of radicals were detected in the ITX/DOPO mixed system during the EPR experiment. These were identified through fitting the EPR spectra obtained after 100 seconds of illumination at 405 nm, including signals potentially attributable to the hydrogen abstraction reaction products of ITX (a_N_ ≈ 14.4 G, a_H_ ≈ 2.6 G, 40.7%), as well as radicals with P content characteristics (a_P_ ≈ 18.7–21.4 G, 59.3%), further supporting the occurrence of hydrogen transfer reactions and proposing the mechanism in Scheme S1.


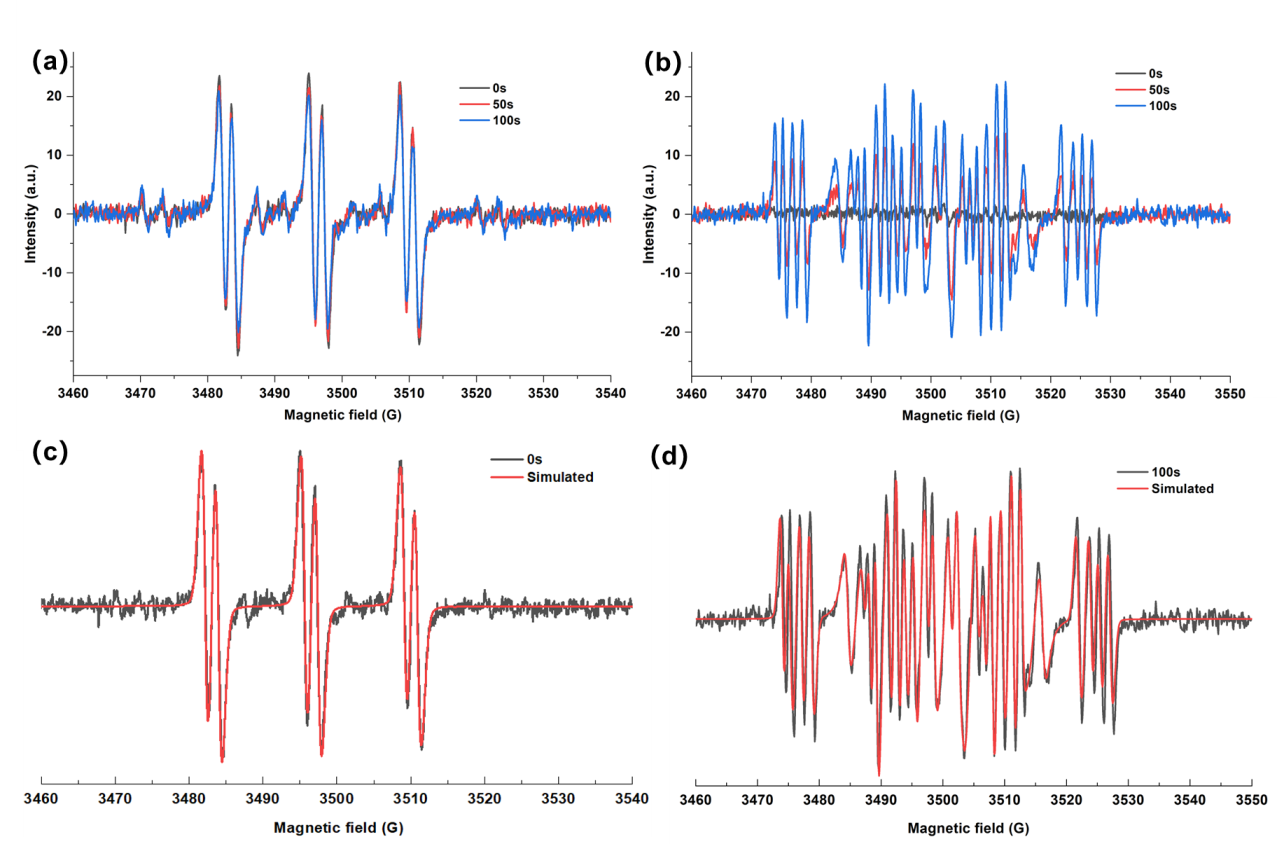


Figure S38. The PBN radical adducts (EPR spectra using *tert*-butylbenzene as a solvent) for (a) DOPO, (b) ITX/DOPO. (c) The simulations at 0s for DOPO. (d) The simulations at 100s for ITX/DOPO.


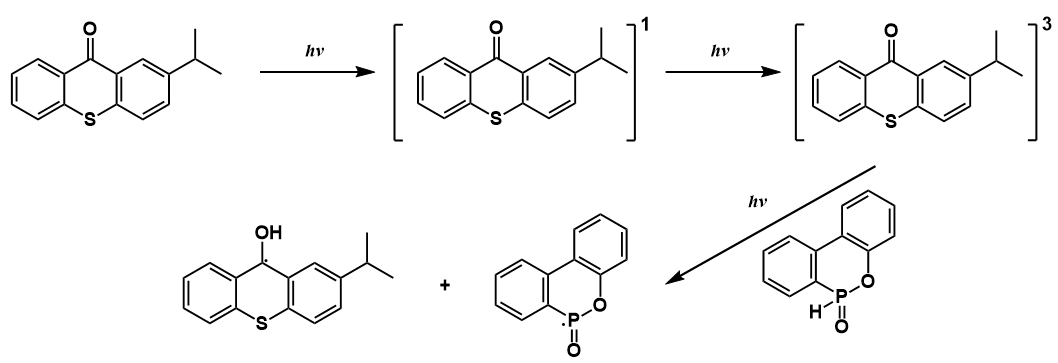


Scheme S1. DOPO as a hydrogen donor and the Type II photoinitiation mechanism of ITX.

Although DOPO does not exhibit polymerization initiation activity in the dark, when combined with ITX to form a two-component photoinitiation system, it may participate in the radical generation process via a dehydrogenation mechanism, thereby enhancing the overall initiation efficiency of the system. The following photophysical chemistry experiment was designed for this purpose. Figure S39a shows the UV-visible absorption spectra of three different photoinitiation systems: ITX, ITX+EDB, and ITX+DOPO in MeCN, as well as their photolysis behavior under LED@385 nm irradiation (Figures S39b–d). As shown in Figure S39a, ITX itself exhibits strong absorption in the 350–450 nm range, with a λmax of approximately 380 nm, making it compatible with commonly used LED light sources. After introducing EDB or DOPO, the absorption profiles of the systems did not undergo significant changes, indicating that these two substances have minimal effects on the absorption bands but may influence the excited-state dynamics. Figures S39b–d illustrate the photolysis processes of ITX and its synergistic systems with EDB and DOPO under 385 nm LED irradiation. When used alone, ITX’s absorption did not show noticeable changes over time. The absorption intensity significantly increases in the 325–375 nm region, indicating that EDB acts as an effective hydrogen donor, promoting the cleavage process of ITX into radicals through electron transfer or hydrogen transfer reactions with the excited state of ITX. This not only enhances the photolysis efficiency of ITX but also indicates that absorption-capable degradation products are generated in this wavelength range, further validating the effective progression of the photoreaction. In the ITX/DOPO system (Figure S39d), although the absorption intensity change is less pronounced than in the EDB system, it still shows a significant enhancement compared to the standalone ITX system. This indicates that DOPO can also participate in the excited state process to some extent, accelerating the photodissociation of ITX through the hydrogen transfer mechanism and thereby improving radical generation efficiency. This result validates the potential of DOPO as a hydrogen donor in Type II photopolymerization systems, holding practical significance for expanding its application in visible light-initiated systems.


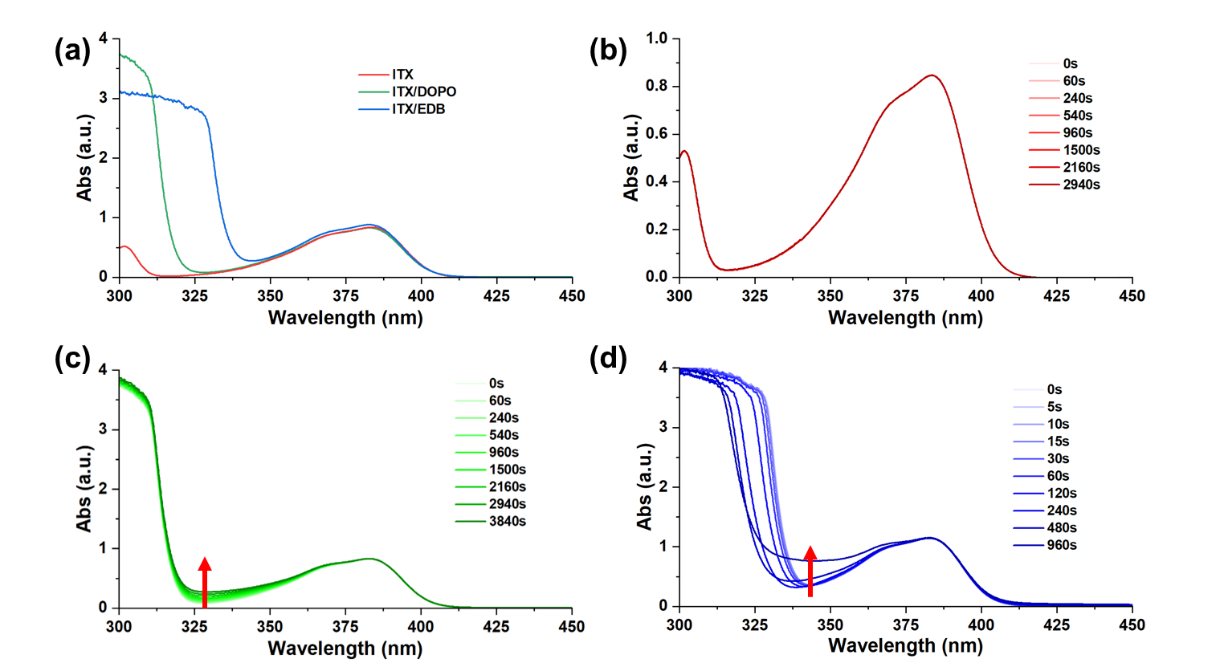


Figure S39. (a) UV-visible absorption spectra of ITX, ITX/EDB, and ITX/DOPO, and photolysis spectra under 385 nm light irradiation (b) ITX (c) ITX/DOPO (d) ITX/EDB.

Figure S40 shows a further comparison of the photopolymerization behavior of the ITX/DOPO and ITX/EDB two-component systems, with polymerization experiments conducted under thick sample conditions (approximately 2 mm, Figure S40a) and thin sample conditions (25 μm, Figure S40b). The results show that the polymerization conversion in samples of different thicknesses indicate that under thick sample conditions (∼2 mm), the polymerization conversion of the ITX/DOPO two-component system is higher than that of the traditional ITX/EDB system and the ITX single-component system, demonstrating excellent initiation efficiency, which suggests that DOPO can effectively participate in the radical initiation process. The order of polymerization was: ITX/EDB > ITX/DOPO > ITX > DOPO. This result indicates that when DOPO is used in conjunction with ITX, although the initial polymerization conversion is lower than that of the traditional ITX/EDB system, it can achieve a higher final conversion under prolonged irradiation, suggesting that the ITX/DOPO system exhibits superior initiation persistence. In thin film samples, although the ITX/DOPO system still outperforms the ITX mono-component system in terms of polymerization conversion, it is slightly inferior to the ITX/EDB combination. Notably, DOPO cannot initiate polymerization reactions when used alone, further indicating that its functionality in Type II initiation systems depends on synergistic interaction with excited-state acceptors such as ITX. The above results validate the effectiveness of DOPO as a hydrogen donor in the Type II mechanism, demonstrating good practical application potential.


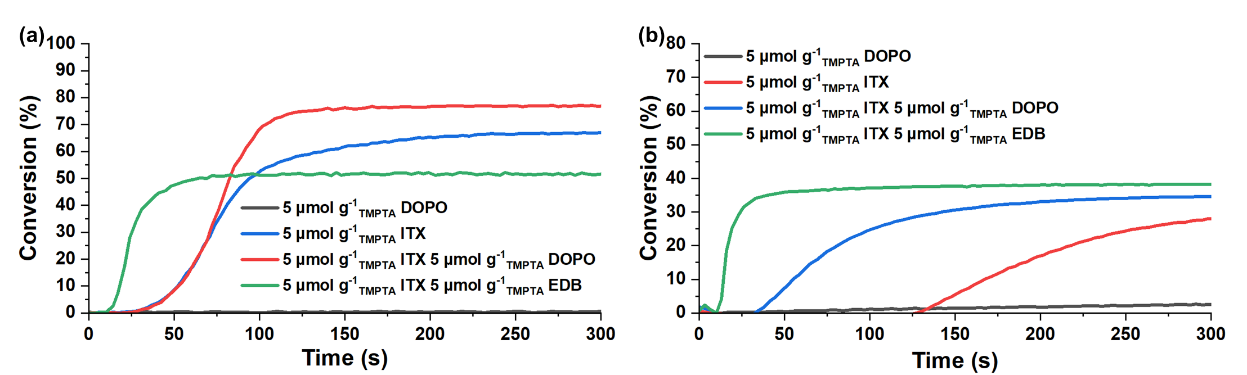


Figure S40. Photopolymerization kinetics of TMPTA with photoinitiators under 405 nm LED irradiation: (a) in a thick sample (∼2 mm) and (b) in a thin film. Irradiation commenced at t = 10 s.

Using 7-(dimethylamino)-4-methyl-2H-chromen-2-one (Couma 3: structure in Figure S41), which has been previously reported by our research group [2], as the photosensitizer, although it exhibited some activity in the previously constructed Type II photoinitiation system, the overall polymerization efficiency was relatively low. To further enhance its initiation performance, this study paired Couma 3 with the traditional hydrogen donor EDB and DOPO to construct two synergistic initiation systems and conducted photopolymerization experiments on PEGDA under LED@405 nm irradiation. The results showed that the Couma 3/DOPO system not only had faster polymerization but also a significantly shorter induction time, outperforming the Couma 3/EDB system (Figure S41). This result indicates that DOPO not only serves as an efficient hydrogen donor in such Type II initiation systems but its unique electronic structure.


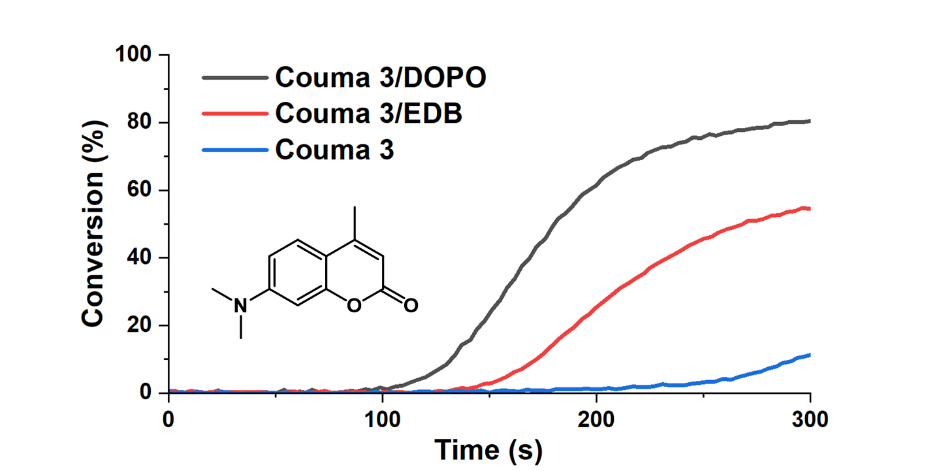


Figure S41. Photopolymerization kinetics of PEGDA with photoinitiators under 405 nm LED irradiation in a thick sample (∼2 mm). Irradiation commenced at t = 10 s.

In summary, DOPO can be introduced as an efficient hydrogen donor into Type II photoinitiation systems, demonstrating excellent synergistic initiation performance. In systems constructed using Couma 3 as the photosensitizer, although this class of aromatic ketone sensitizers has previously been reported to have limited polymerization efficiency, when paired with DOPO, not only is the induction period significantly shortened, but the polymerization is also significantly enhanced, significantly outperforming the traditional hydrogen donor EDB. In systems based on ITX, DOPO also exhibits good synergistic enhancement effects under thick film curing conditions, effectively promoting radical generation. These results indicate that DOPO plays a hydrogen donor role in Type II photoinitiation.

**Cytotoxicity experiments**

For cell morphology, proliferation and time-lapse imaging, a Holomonitor® M4 phase holographic microscope (PHI AB, Lund, Sweden) was used with a 10 times phase contrast objective, placed in an incubator at 37°C and 5% CO_2_. The C3H10 T1/2 cells lines were seeded in a 6 well plate (Sarstedt AG & Co. KG, Germany) the day before the test to attach at 20000 cells/well in Dulbecco’s modified eagle medium (DMEM) 10 vol% fetal bovine serum (FBS). The concentration used for each PI is 50µM. The medium with this concentration of PI was replace just before to start the acquisition. The lid of the 6-well plate was replaced with the HoloLid^®^ 71120 for 6-well plate (PHI AB) and then placed on the motorized stage of the HoloMonitor^®^ M4 in the incubator. Images were acquired at 10 locations per well, at 30 minutes’ intervals for 20 hours.


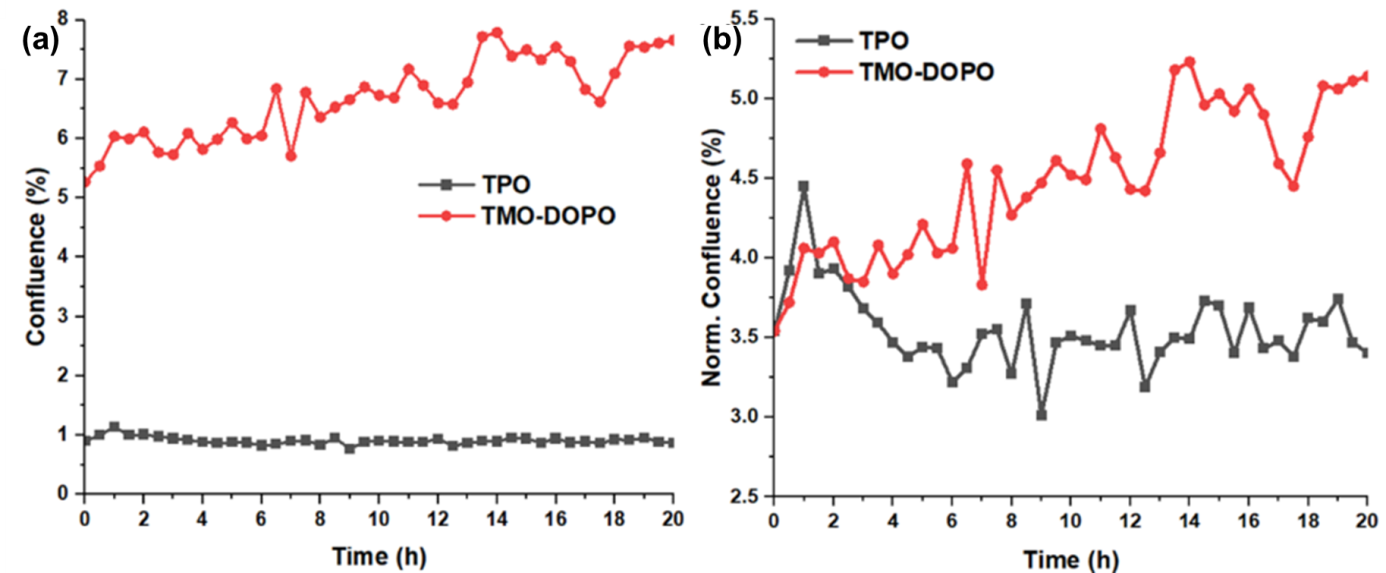


Figure S42. (a) Confluences of cells per well: TPO vs. TMO-DOPO groups. (b) Normalized confluences of cells per well comparison: TPO vs. TMO-DOPO groups.

**References**

1. C. Dietlin, T. T. Trinh, S. Schweizer, B. Graff, F. MorletSavary, P. Noirot, J. Lalevée, Macromolecules 2019, 52, 7886–7893.
2. T. Gao, F. Morlet-Savary, Bernadette Graff, F. Dumur, J. Zhang, Pu Xiao, J. Lalevée, Polym. Chem., 2024,15, 2416-2427.
